# Search for ${\text {Z}{}{}} {\text {Z}{}{}}$ and ${\text {Z}{}{}} {\text {H}{}{}}$ production in the ${\text {b}{}{}} {\bar{{\text {b}{}{}}}{}{}} {\text {b}{}{}} {\bar{{\text {b}{}{}}}{}{}}$ final state using proton-proton collisions at $\sqrt{s}=13\,\text {Te}\hspace{-.08em}\text {V}$

**DOI:** 10.1140/epjc/s10052-024-13021-z

**Published:** 2024-07-19

**Authors:** CMS Collaboration, A. Hayrapetyan, A. Tumasyan, W. Adam, J. W. Andrejkovic, T. Bergauer, S. Chatterjee, K. Damanakis, M. Dragicevic, P. S. Hussain, M. Jeitler, N. Krammer, A. Li, D. Liko, I. Mikulec, J. Schieck, R. Schöfbeck, D. Schwarz, M. Sonawane, S. Templ, W. Waltenberger, C.-E. Wulz, M. R. Darwish, T. Janssen, P. Van Mechelen, E. S. Bols, J. D’Hondt, S. Dansana, A. De Moor, M. Delcourt, S. Lowette, I. Makarenko, D. Müller, S. Tavernier, M. Tytgat, G. P. Van Onsem, S. Van Putte, D. Vannerom, B. Clerbaux, A. K. Das, G. De Lentdecker, H. Evard, L. Favart, P. Gianneios, D. Hohov, J. Jaramillo, A. Khalilzadeh, F. A. Khan, K. Lee, M. Mahdavikhorrami, A. Malara, S. Paredes, L. Thomas, M. Vanden Bemden, C. Vander Velde, P. Vanlaer, M. De Coen, D. Dobur, Y. Hong, J. Knolle, L. Lambrecht, G. Mestdach, K. Mota Amarilo, C. Rendón, A. Samalan, K. Skovpen, N. Van Den Bossche, J. van der Linden, L. Wezenbeek, A. Benecke, A. Bethani, G. Bruno, C. Caputo, C. Delaere, I. S. Donertas, A. Giammanco, Sa. Jain, V. Lemaitre, J. Lidrych, P. Mastrapasqua, T. T. Tran, S. Wertz, G. A. Alves, E. Coelho, C. Hensel, T. Menezes De Oliveira, A. Moraes, P. Rebello Teles, M. Soeiro, W. L. Aldá Júnior, M. Alves Gallo Pereira, M. Barroso Ferreira Filho, H. Brandao Malbouisson, W. Carvalho, J. Chinellato, E. M. Da Costa, G. G. Da Silveira, D. De Jesus Damiao, S. Fonseca De Souza, R. Gomes De Souza, J. Martins, C. Mora Herrera, L. Mundim, H. Nogima, J. P. Pinheiro, A. Santoro, A. Sznajder, M. Thiel, A. Vilela Pereira, C. A. Bernardes, L. Calligaris, T. R. Fernandez Perez Tomei, E. M. Gregores, P. G. Mercadante, S. F. Novaes, B. Orzari, Sandra S. Padula, A. Aleksandrov, G. Antchev, R. Hadjiiska, P. Iaydjiev, M. Misheva, M. Shopova, G. Sultanov, A. Dimitrov, L. Litov, B. Pavlov, P. Petkov, A. Petrov, E. Shumka, S. Keshri, S. Thakur, T. Cheng, T. Javaid, L. Yuan, Z. Hu, J. Liu, K. Yi, G. M. Chen, H. S. Chen, M. Chen, F. Iemmi, C. H. Jiang, A. Kapoor, H. Liao, Z. -A. Liu, R. Sharma, J. N. Song, J. Tao, C. Wang, J. Wang, Z. Wang, H. Zhang, A. Agapitos, Y. Ban, A. Levin, C. Li, Q. Li, Y. Mao, S. J. Qian, X. Sun, D. Wang, H. Yang, L. Zhang, C. Zhou, Z. You, K. Jaffel, N. Lu, G. Bauer, X. Gao, Z. Lin, C. Lu, M. Xiao, C. Avila, D. A. Barbosa Trujillo, A. Cabrera, C. Florez, J. Fraga, J. A. Reyes Vega, J. Mejia Guisao, F. Ramirez, M. Rodriguez, J. D. Ruiz Alvarez, D. Giljanovic, N. Godinovic, D. Lelas, A. Sculac, M. Kovac, T. Sculac, P. Bargassa, V. Brigljevic, B. K. Chitroda, D. Ferencek, K. Jakovcic, S. Mishra, A. Starodumov, T. Susa, A. Attikis, K. Christoforou, A. Hadjiagapiou, S. Konstantinou, J. Mousa, C. Nicolaou, L. Paizanos, F. Ptochos, P. A. Razis, H. Rykaczewski, H. Saka, A. Stepennov, M. Finger, M. Finger, A. Kveton, E. Ayala, E. Carrera Jarrin, H. Abdalla, Y. Assran, M. Abdullah Al-Mashad, M. A. Mahmoud, K. Ehataht, M. Kadastik, T. Lange, S. Nandan, C. Nielsen, J. Pata, M. Raidal, L. Tani, C. Veelken, H. Kirschenmann, K. Osterberg, M. Voutilainen, S. Bharthuar, E. Brücken, F. Garcia, K. T. S. Kallonen, R. Kinnunen, T. Lampén, K. Lassila-Perini, S. Lehti, T. Lindén, L. Martikainen, M. Myllymäki, M. m. Rantanen, H. Siikonen, E. Tuominen, J. Tuominiemi, P. Luukka, H. Petrow, M. Besancon, F. Couderc, M. Dejardin, D. Denegri, J. L. Faure, F. Ferri, S. Ganjour, P. Gras, G. Hamel de Monchenault, V. Lohezic, J. Malcles, F. Orlandi, L. Portales, J. Rander, A. Rosowsky, M. Ö. Sahin, A. Savoy-Navarro, P. Simkina, M. Titov, M. Tornago, F. Beaudette, A. Buchot Perraguin, P. Busson, A. Cappati, C. Charlot, M. Chiusi, F. Damas, O. Davignon, A. De Wit, I. T. Ehle, B. A. Fontana Santos Alves, S. Ghosh, A. Gilbert, R. Granier de Cassagnac, A. Hakimi, B. Harikrishnan, L. Kalipoliti, G. Liu, J. Motta, M. Nguyen, C. Ochando, R. Salerno, J. B. Sauvan, Y. Sirois, A. Tarabini, E. Vernazza, A. Zabi, A. Zghiche, J. -L. Agram, J. Andrea, D. Apparu, D. Bloch, J. -M. Brom, E. C. Chabert, C. Collard, S. Falke, U. Goerlach, C. Grimault, R. Haeberle, A. -C. Le Bihan, M. Meena, G. Saha, M. A. Sessini, P. Van Hove, S. Beauceron, B. Blancon, G. Boudoul, N. Chanon, D. Contardo, P. Depasse, C. Dozen, H. El Mamouni, J. Fay, S. Gascon, M. Gouzevitch, C. Greenberg, G. Grenier, B. Ille, I. B. Laktineh, M. Lethuillier, L. Mirabito, S. Perries, A. Purohit, M. Vander Donckt, P. Verdier, J. Xiao, G. Adamov, I. Lomidze, Z. Tsamalaidze, V. Botta, L. Feld, K. Klein, M. Lipinski, D. Meuser, A. Pauls, N. Röwert, M. Teroerde, S. Diekmann, A. Dodonova, N. Eich, D. Eliseev, F. Engelke, J. Erdmann, M. Erdmann, P. Fackeldey, B. Fischer, T. Hebbeker, K. Hoepfner, F. Ivone, A. Jung, M. y. Lee, F. Mausolf, M. Merschmeyer, A. Meyer, S. Mukherjee, D. Noll, F. Nowotny, A. Pozdnyakov, Y. Rath, W. Redjeb, F. Rehm, H. Reithler, U. Sarkar, V. Sarkisovi, A. Schmidt, A. Sharma, J. L. Spah, A. Stein, F. Torres Da Silva De Araujo, S. Wiedenbeck, S. Zaleski, C. Dziwok, G. Flügge, W. Haj Ahmad, T. Kress, A. Nowack, O. Pooth, A. Stahl, T. Ziemons, A. Zotz, H. Aarup Petersen, M. Aldaya Martin, J. Alimena, S. Amoroso, Y. An, S. Baxter, M. Bayatmakou, H. Becerril Gonzalez, O. Behnke, A. Belvedere, S. Bhattacharya, F. Blekman, K. Borras, A. Campbell, A. Cardini, C. Cheng, F. Colombina, S. Consuegra Rodríguez, G. Correia Silva, M. De Silva, G. Eckerlin, D. Eckstein, L. I. Estevez Banos, O. Filatov, E. Gallo, A. Geiser, A. Giraldi, V. Guglielmi, M. Guthoff, A. Hinzmann, A. Jafari, L. Jeppe, B. Kaech, M. Kasemann, C. Kleinwort, R. Kogler, M. Komm, D. Krücker, W. Lange, D. Leyva Pernia, K. Lipka, W. Lohmann, F. Lorkowski, R. Mankel, I. -A. Melzer-Pellmann, M. Mendizabal Morentin, A. B. Meyer, G. Milella, A. Mussgiller, L. P. Nair, A. Nürnberg, Y. Otarid, J. Park, D. Pérez Adán, E. Ranken, A. Raspereza, D. Rastorguev, B. Ribeiro Lopes, J. Rübenach, A. Saggio, M. Scham, S. Schnake, P. Schütze, C. Schwanenberger, D. Selivanova, K. Sharko, M. Shchedrolosiev, R. E. Sosa Ricardo, D. Stafford, F. Vazzoler, A. Ventura Barroso, R. Walsh, Q. Wang, Y. Wen, K. Wichmann, L. Wiens, C. Wissing, Y. Yang, A. Zimermmane Castro Santos, A. Albrecht, S. Albrecht, M. Antonello, S. Bein, L. Benato, S. Bollweg, M. Bonanomi, P. Connor, K. El Morabit, Y. Fischer, E. Garutti, A. Grohsjean, J. Haller, H. R. Jabusch, G. Kasieczka, P. Keicher, R. Klanner, W. Korcari, T. Kramer, V. Kutzner, F. Labe, J. Lange, A. Lobanov, C. Matthies, L. Moureaux, M. Mrowietz, A. Nigamova, Y. Nissan, A. Paasch, K. J. Pena Rodriguez, T. Quadfasel, B. Raciti, M. Rieger, D. Savoiu, J. Schindler, P. Schleper, M. Schröder, J. Schwandt, M. Sommerhalder, H. Stadie, G. Steinbrück, A. Tews, M. Wolf, S. Brommer, M. Burkart, E. Butz, T. Chwalek, A. Dierlamm, A. Droll, N. Faltermann, M. Giffels, A. Gottmann, F. Hartmann, R. Hofsaess, M. Horzela, U. Husemann, J. Kieseler, M. Klute, R. Koppenhöfer, J. M. Lawhorn, M. Link, A. Lintuluoto, B. Maier, S. Maier, S. Mitra, M. Mormile, Th. Müller, M. Neukum, M. Oh, E. Pfeffer, M. Presilla, G. Quast, K. Rabbertz, B. Regnery, N. Shadskiy, I. Shvetsov, H. J. Simonis, M. Toms, N. Trevisani, R. F. Von Cube, M. Wassmer, S. Wieland, F. Wittig, R. Wolf, X. Zuo, G. Anagnostou, G. Daskalakis, A. Kyriakis, A. Papadopoulos, A. Stakia, P. Kontaxakis, G. Melachroinos, Z. Painesis, A. Panagiotou, I. Papavergou, I. Paraskevas, N. Saoulidou, K. Theofilatos, E. Tziaferi, K. Vellidis, I. Zisopoulos, G. Bakas, T. Chatzistavrou, G. Karapostoli, K. Kousouris, I. Papakrivopoulos, E. Siamarkou, G. Tsipolitis, A. Zacharopoulou, K. Adamidis, I. Bestintzanos, I. Evangelou, C. Foudas, C. Kamtsikis, P. Katsoulis, P. Kokkas, P. G. Kosmoglou Kioseoglou, N. Manthos, I. Papadopoulos, J. Strologas, M. Bartók, C. Hajdu, D. Horvath, K. Márton, A. J. Rádl, F. Sikler, V. Veszpremi, M. Csanád, K. Farkas, M. M. A. Gadallah, Á. Kadlecsik, P. Major, K. Mandal, G. Pásztor, G. I. Veres, P. Raics, B. Ujvari, G. Zilizi, G. Bencze, S. Czellar, J. Molnar, Z. Szillasi, T. Csorgo, F. Nemes, T. Novak, J. Babbar, S. Bansal, S. B. Beri, V. Bhatnagar, G. Chaudhary, S. Chauhan, N. Dhingra, A. Kaur, A. Kaur, H. Kaur, M. Kaur, S. Kumar, K. Sandeep, T. Sheokand, J. B. Singh, A. Singla, A. Ahmed, A. Bhardwaj, A. Chhetri, B. C. Choudhary, A. Kumar, A. Kumar, M. Naimuddin, K. Ranjan, S. Saumya, S. Baradia, S. Barman, S. Bhattacharya, S. Dutta, S. Dutta, S. Sarkar, M. M. Ameen, P. K. Behera, S. C. Behera, S. Chatterjee, P. Jana, P. Kalbhor, J. R. Komaragiri, D. Kumar, P. R. Pujahari, N. R. Saha, A. Sharma, A. K. Sikdar, S. Verma, S. Dugad, M. Kumar, G. B. Mohanty, P. Suryadevara, A. Bala, S. Banerjee, R. M. Chatterjee, R. K. Dewanjee, M. Guchait, Sh. Jain, A. Jaiswal, S. Kumar, G. Majumder, K. Mazumdar, S. Parolia, A. Thachayath, S. Bahinipati, C. Kar, D. Maity, P. Mal, T. Mishra, V. K. Muraleedharan Nair Bindhu, K. Naskar, A. Nayak, P. Sadangi, S. K. Swain, S. Varghese, D. Vats, S. Acharya, A. Alpana, S. Dube, B. Gomber, P. Hazarika, B. Kansal, A. Laha, B. Sahu, S. Sharma, K. Y. Vaish, H. Bakhshiansohi, E. Khazaie, M. Zeinali, S. Bashiri, S. Chenarani, S. M. Etesami, M. Khakzad, M. Mohammadi Najafabadi, S. Tizchang, M. Grunewald, M. Abbrescia, R. Aly, A. Colaleo, D. Creanza, B. D’Anzi, N. De Filippis, M. De Palma, A. Di Florio, W. Elmetenawee, L. Fiore, G. Iaselli, M. Louka, G. Maggi, M. Maggi, I. Margjeka, V. Mastrapasqua, S. My, S. Nuzzo, A. Pellecchia, A. Pompili, G. Pugliese, R. Radogna, G. Ramirez-Sanchez, D. Ramos, A. Ranieri, L. Silvestris, F. M. Simone, Ü. Sözbilir, A. Stamerra, R. Venditti, P. Verwilligen, A. Zaza, G. Abbiendi, C. Battilana, D. Bonacorsi, L. Borgonovi, P. Capiluppi, A. Castro, F. R. Cavallo, M. Cuffiani, G. M. Dallavalle, T. Diotalevi, F. Fabbri, A. Fanfani, D. Fasanella, P. Giacomelli, L. Giommi, C. Grandi, L. Guiducci, S. Lo Meo, L. Lunerti, S. Marcellini, G. Masetti, F. L. Navarria, A. Perrotta, F. Primavera, A. M. Rossi, T. Rovelli, G. P. Siroli, S. Costa, A. Di Mattia, R. Potenza, A. Tricomi, C. Tuve, P. Assiouras, G. Barbagli, G. Bardelli, B. Camaiani, A. Cassese, R. Ceccarelli, V. Ciulli, C. Civinini, R. D’Alessandro, E. Focardi, T. Kello, G. Latino, P. Lenzi, M. Lizzo, M. Meschini, S. Paoletti, A. Papanastassiou, G. Sguazzoni, L. Viliani, L. Benussi, S. Bianco, S. Meola, D. Piccolo, P. Chatagnon, F. Ferro, E. Robutti, S. Tosi, A. Benaglia, G. Boldrini, F. Brivio, F. Cetorelli, F. De Guio, M. E. Dinardo, P. Dini, S. Gennai, R. Gerosa, A. Ghezzi, P. Govoni, L. Guzzi, M. T. Lucchini, M. Malberti, S. Malvezzi, A. Massironi, D. Menasce, L. Moroni, M. Paganoni, S. Palluotto, D. Pedrini, B. S. Pinolini, G. Pizzati, S. Ragazzi, T. Tabarelli de Fatis, S. Buontempo, A. Cagnotta, F. Carnevali, N. Cavallo, F. Fabozzi, A. O. M. Iorio, L. Lista, P. Paolucci, B. Rossi, C. Sciacca, R. Ardino, P. Azzi, N. Bacchetta, M. Benettoni, D. Bisello, P. Bortignon, G. Bortolato, A. Bragagnolo, A. C. M. Bulla, R. Carlin, P. Checchia, T. Dorigo, U. Gasparini, E. Lusiani, M. Margoni, F. Marini, A. T. Meneguzzo, M. Migliorini, J. Pazzini, P. Ronchese, R. Rossin, F. Simonetto, G. Strong, M. Tosi, A. Triossi, S. Ventura, M. Zanetti, P. Zotto, A. Zucchetta, G. Zumerle, S. Abu Zeid, C. Aimè, A. Braghieri, S. Calzaferri, D. Fiorina, P. Montagna, V. Re, C. Riccardi, P. Salvini, I. Vai, P. Vitulo, S. Ajmal, G. M. Bilei, D. Ciangottini, L. Fanò, M. Magherini, V. Mariani, M. Menichelli, F. Moscatelli, A. Rossi, A. Santocchia, D. Spiga, T. Tedeschi, P. Asenov, P. Azzurri, G. Bagliesi, R. Bhattacharya, L. Bianchini, T. Boccali, E. Bossini, D. Bruschini, R. Castaldi, M. A. Ciocci, M. Cipriani, V. D’Amante, R. Dell’Orso, S. Donato, A. Giassi, F. Ligabue, D. Matos Figueiredo, A. Messineo, M. Musich, F. Palla, A. Rizzi, G. Rolandi, S. Roy Chowdhury, T. Sarkar, A. Scribano, P. Spagnolo, R. Tenchini, G. Tonelli, N. Turini, F. Vaselli, A. Venturi, P. G. Verdini, C. Baldenegro Barrera, P. Barria, C. Basile, M. Campana, F. Cavallari, L. Cunqueiro Mendez, D. Del Re, E. Di Marco, M. Diemoz, F. Errico, E. Longo, P. Meridiani, J. Mijuskovic, G. Organtini, F. Pandolfi, R. Paramatti, C. Quaranta, S. Rahatlou, C. Rovelli, F. Santanastasio, L. Soffi, N. Amapane, R. Arcidiacono, S. Argiro, M. Arneodo, N. Bartosik, R. Bellan, A. Bellora, C. Biino, C. Borca, N. Cartiglia, M. Costa, R. Covarelli, N. Demaria, L. Finco, M. Grippo, B. Kiani, F. Legger, F. Luongo, C. Mariotti, L. Markovic, S. Maselli, A. Mecca, E. Migliore, M. Monteno, R. Mulargia, M. M. Obertino, G. Ortona, L. Pacher, N. Pastrone, M. Pelliccioni, M. Ruspa, F. Siviero, V. Sola, A. Solano, A. Staiano, C. Tarricone, D. Trocino, G. Umoret, E. Vlasov, R. White, S. Belforte, V. Candelise, M. Casarsa, F. Cossutti, K. De Leo, G. Della Ricca, S. Dogra, J. Hong, C. Huh, B. Kim, D. H. Kim, J. Kim, H. Lee, S. W. Lee, C. S. Moon, Y. D. Oh, M. S. Ryu, S. Sekmen, Y. C. Yang, M. S. Kim, G. Bak, P. Gwak, H. Kim, D. H. Moon, E. Asilar, J. Choi, D. Kim, T. J. Kim, J. A. Merlin, S. Choi, S. Han, B. Hong, K. Lee, K. S. Lee, S. Lee, J. Park, S. K. Park, J. Yoo, J. Goh, S. Yang, H. S. Kim, Y. Kim, S. Lee, J. Almond, J. H. Bhyun, J. Choi, W. Jun, J. Kim, S. Ko, H. Kwon, H. Lee, J. Lee, J. Lee, B. H. Oh, S. B. Oh, H. Seo, U. K. Yang, I. Yoon, W. Jang, D. Y. Kang, Y. Kang, S. Kim, B. Ko, J. S. H. Lee, Y. Lee, I. C. Park, Y. Roh, I. J. Watson, S. Ha, H. D. Yoo, M. Choi, M. R. Kim, H. Lee, Y. Lee, I. Yu, T. Beyrouthy, K. Dreimanis, A. Gaile, G. Pikurs, A. Potrebko, M. Seidel, N. R. Strautnieks, M. Ambrozas, A. Juodagalvis, A. Rinkevicius, G. Tamulaitis, N. Bin Norjoharuddeen, I. Yusuff, Z. Zolkapli, J. F. Benitez, A. Castaneda Hernandez, H. A. Encinas Acosta, L. G. Gallegos Maríñez, M. León Coello, J. A. Murillo Quijada, A. Sehrawat, L. Valencia Palomo, G. Ayala, H. Castilla-Valdez, H. Crotte Ledesma, E. De La Cruz-Burelo, I. Heredia-De La Cruz, R. Lopez-Fernandez, C. A. Mondragon Herrera, A. Sánchez Hernández, C. Oropeza Barrera, M. Ramírez García, I. Bautista, I. Pedraza, H. A. Salazar Ibarguen, C. Uribe Estrada, I. Bubanja, N. Raicevic, P. H. Butler, A. Ahmad, M. I. Asghar, A. Awais, M. I. M. Awan, H. R. Hoorani, W. A. Khan, V. Avati, L. Grzanka, M. Malawski, H. Bialkowska, M. Bluj, B. Boimska, M. Górski, M. Kazana, M. Szleper, P. Zalewski, K. Bunkowski, K. Doroba, A. Kalinowski, M. Konecki, J. Krolikowski, A. Muhammad, K. Pozniak, W. Zabolotny, M. Araujo, D. Bastos, C. Beirão Da Cruz E Silva, A. Boletti, M. Bozzo, T. Camporesi, G. Da Molin, P. Faccioli, M. Gallinaro, J. Hollar, N. Leonardo, T. Niknejad, A. Petrilli, M. Pisano, J. Seixas, J. Varela, J. W. Wulff, P. Adzic, P. Milenovic, M. Dordevic, J. Milosevic, V. Rekovic, M. Aguilar-Benitez, J. Alcaraz Maestre, Cristina F. Bedoya, Oliver M. Carretero, M. Cepeda, M. Cerrada, N. Colino, B. De La Cruz, A. Delgado Peris, A. Escalante Del Valle, D. Fernández Del Val, J. P. Fernández Ramos, J. Flix, M. C. Fouz, O. Gonzalez Lopez, S. Goy Lopez, J. M. Hernandez, M. I. Josa, D. Moran, C. M. Morcillo Perez, Á. Navarro Tobar, C. Perez Dengra, A. Pérez-Calero Yzquierdo, J. Puerta Pelayo, I. Redondo, D. D. Redondo Ferrero, L. Romero, S. Sánchez Navas, L. Urda Gómez, J. Vazquez Escobar, C. Willmott, J. F. de Trocóniz, B. Alvarez Gonzalez, J. Cuevas, J. Fernandez Menendez, S. Folgueras, I. Gonzalez Caballero, J. R. González Fernández, P. Leguina, E. Palencia Cortezon, C. Ramón Álvarez, V. Rodríguez Bouza, A. Soto Rodríguez, A. Trapote, C. Vico Villalba, P. Vischia, S. Bhowmik, S. Blanco Fernández, J. A. Brochero Cifuentes, I. J. Cabrillo, A. Calderon, J. Duarte Campderros, M. Fernandez, G. Gomez, C. Lasaosa García, R. Lopez Ruiz, C. Martinez Rivero, P. Martinez Ruiz del Arbol, F. Matorras, P. Matorras Cuevas, E. Navarrete Ramos, J. Piedra Gomez, L. Scodellaro, I. Vila, J. M. Vizan Garcia, M. K. Jayananda, B. Kailasapathy, D. U. J. Sonnadara, D. D. C. Wickramarathna, W. G. D. Dharmaratna, K. Liyanage, N. Perera, N. Wickramage, D. Abbaneo, C. Amendola, E. Auffray, G. Auzinger, J. Baechler, D. Barney, A. Bermúdez Martínez, M. Bianco, B. Bilin, A. A. Bin Anuar, A. Bocci, C. Botta, E. Brondolin, C. Caillol, G. Cerminara, N. Chernyavskaya, D. d’Enterria, A. Dabrowski, A. David, A. De Roeck, M. M. Defranchis, M. Deile, M. Dobson, L. Forthomme, G. Franzoni, W. Funk, S. Giani, D. Gigi, K. Gill, F. Glege, L. Gouskos, M. Haranko, J. Hegeman, B. Huber, V. Innocente, T. James, P. Janot, O. Kaluzinska, S. Laurila, P. Lecoq, E. Leutgeb, C. Lourenço, L. Malgeri, M. Mannelli, A. C. Marini, M. Matthewman, A. Mehta, F. Meijers, S. Mersi, E. Meschi, V. Milosevic, F. Monti, F. Moortgat, M. Mulders, I. Neutelings, S. Orfanelli, F. Pantaleo, G. Petrucciani, A. Pfeiffer, M. Pierini, D. Piparo, H. Qu, D. Rabady, M. Rovere, H. Sakulin, S. Scarfi, C. Schwick, M. Selvaggi, A. Sharma, K. Shchelina, P. Silva, P. Sphicas, A. G. Stahl Leiton, A. Steen, S. Summers, D. Treille, P. Tropea, A. Tsirou, D. Walter, J. Wanczyk, J. Wang, S. Wuchterl, P. Zehetner, P. Zejdl, W. D. Zeuner, T. Bevilacqua, L. Caminada, A. Ebrahimi, W. Erdmann, R. Horisberger, Q. Ingram, H. C. Kaestli, D. Kotlinski, C. Lange, M. Missiroli, L. Noehte, T. Rohe, T. K. Aarrestad, K. Androsov, M. Backhaus, G. Bonomelli, A. Calandri, C. Cazzaniga, K. Datta, A. De Cosa, G. Dissertori, M. Dittmar, M. Donegà, F. Eble, M. Galli, K. Gedia, F. Glessgen, C. Grab, N. Härringer, T. G. Harte, D. Hits, W. Lustermann, A. -M. Lyon, R. A. Manzoni, M. Marchegiani, L. Marchese, C. Martin Perez, A. Mascellani, F. Nessi-Tedaldi, F. Pauss, V. Perovic, S. Pigazzini, C. Reissel, T. Reitenspiess, B. Ristic, F. Riti, R. Seidita, J. Steggemann, D. Valsecchi, R. Wallny, C. Amsler, P. Bärtschi, M. F. Canelli, K. Cormier, J. K. Heikkilä, M. Huwiler, W. Jin, A. Jofrehei, B. Kilminster, S. Leontsinis, S. P. Liechti, A. Macchiolo, P. Meiring, U. Molinatti, A. Reimers, P. Robmann, S. Sanchez Cruz, M. Senger, E. Shokr, F. Stäger, Y. Takahashi, R. Tramontano, C. Adloff, D. Bhowmik, C. M. Kuo, W. Lin, P. K. Rout, P. C. Tiwari, S. S. Yu, L. Ceard, Y. Chao, K. F. Chen, P. s. Chen, Z. g. Chen, A. De Iorio, W. -S. Hou, T. h. Hsu, Y. w. Kao, S. Karmakar, R. Khurana, G. Kole, Y. y. Li, R. -S. Lu, E. Paganis, X. f. Su, J. Thomas-Wilsker, L. s. Tsai, H. y. Wu, E. Yazgan, C. Asawatangtrakuldee, N. Srimanobhas, V. Wachirapusitanand, D. Agyel, F. Boran, Z. S. Demiroglu, F. Dolek, I. Dumanoglu, E. Eskut, Y. Guler, E. Gurpinar Guler, C. Isik, O. Kara, A. Kayis Topaksu, U. Kiminsu, G. Onengut, K. Ozdemir, A. Polatoz, B. Tali, U. G. Tok, S. Turkcapar, E. Uslan, I. S. Zorbakir, G. Sokmen, M. Yalvac, B. Akgun, I. O. Atakisi, E. Gülmez, M. Kaya, O. Kaya, S. Tekten, A. Cakir, K. Cankocak, G. G. Dincer, Y. Komurcu, S. Sen, O. Aydilek, S. Cerci, V. Epshteyn, B. Hacisahinoglu, I. Hos, B. Kaynak, S. Ozkorucuklu, O. Potok, H. Sert, C. Simsek, C. Zorbilmez, B. Isildak, D. Sunar Cerci, A. Boyaryntsev, B. Grynyov, L. Levchuk, D. Anthony, J. J. Brooke, A. Bundock, F. Bury, E. Clement, D. Cussans, H. Flacher, M. Glowacki, J. Goldstein, H. F. Heath, M. -L. Holmberg, L. Kreczko, S. Paramesvaran, L. Robertshaw, S. Seif El Nasr-Storey, V. J. Smith, N. Stylianou, K. Walkingshaw Pass, A. H. Ball, K. W. Bell, A. Belyaev, C. Brew, R. M. Brown, D. J. A. Cockerill, C. Cooke, K. V. Ellis, K. Harder, S. Harper, J. Linacre, K. Manolopoulos, D. M. Newbold, E. Olaiya, D. Petyt, T. Reis, A. R. Sahasransu, G. Salvi, T. Schuh, C. H. Shepherd-Themistocleous, I. R. Tomalin, T. Williams, R. Bainbridge, P. Bloch, C. E. Brown, O. Buchmuller, V. Cacchio, C. A. Carrillo Montoya, G. S. Chahal, D. Colling, J. S. Dancu, I. Das, P. Dauncey, G. Davies, J. Davies, M. Della Negra, S. Fayer, G. Fedi, G. Hall, M. H. Hassanshahi, A. Howard, G. Iles, M. Knight, J. Langford, J. León Holgado, L. Lyons, A. -M. Magnan, S. Malik, M. Mieskolainen, J. Nash, M. Pesaresi, B. C. Radburn-Smith, A. Richards, A. Rose, K. Savva, C. Seez, R. Shukla, A. Tapper, K. Uchida, G. P. Uttley, L. H. Vage, T. Virdee, M. Vojinovic, N. Wardle, D. Winterbottom, K. Coldham, J. E. Cole, A. Khan, P. Kyberd, I. D. Reid, S. Abdullin, A. Brinkerhoff, B. Caraway, E. Collins, J. Dittmann, K. Hatakeyama, J. Hiltbrand, B. McMaster, S. Sawant, C. Sutantawibul, J. Wilson, R. Bartek, A. Dominguez, C. Huerta Escamilla, A. E. Simsek, R. Uniyal, A. M. Vargas Hernandez, B. Bam, R. Chudasama, S. I. Cooper, S. V. Gleyzer, C. U. Perez, P. Rumerio, E. Usai, R. Yi, A. Akpinar, D. Arcaro, C. Cosby, Z. Demiragli, C. Erice, C. Fangmeier, C. Fernandez Madrazo, E. Fontanesi, D. Gastler, F. Golf, S. Jeon, I. Reed, J. Rohlf, K. Salyer, D. Sperka, D. Spitzbart, I. Suarez, A. Tsatsos, S. Yuan, A. G. Zecchinelli, G. Benelli, X. Coubez, D. Cutts, M. Hadley, U. Heintz, J. M. Hogan, T. Kwon, G. Landsberg, K. T. Lau, D. Li, J. Luo, S. Mondal, M. Narain, N. Pervan, S. Sagir, F. Simpson, M. Stamenkovic, N. Venkatasubramanian, X. Yan, W. Zhang, S. Abbott, J. Bonilla, C. Brainerd, R. Breedon, H. Cai, M. Calderon De La Barca Sanchez, M. Chertok, M. Citron, J. Conway, P. T. Cox, R. Erbacher, F. Jensen, O. Kukral, G. Mocellin, M. Mulhearn, D. Pellett, W. Wei, Y. Yao, F. Zhang, M. Bachtis, R. Cousins, A. Datta, G. Flores Avila, J. Hauser, M. Ignatenko, M. A. Iqbal, T. Lam, E. Manca, A. Nunez Del Prado, D. Saltzberg, V. Valuev, R. Clare, J. W. Gary, M. Gordon, G. Hanson, W. Si, S. Wimpenny, J. G. Branson, S. Cittolin, S. Cooperstein, D. Diaz, J. Duarte, L. Giannini, J. Guiang, R. Kansal, V. Krutelyov, R. Lee, J. Letts, M. Masciovecchio, F. Mokhtar, S. Mukherjee, M. Pieri, M. Quinnan, B. V. Sathia Narayanan, V. Sharma, M. Tadel, E. Vourliotis, F. Würthwein, Y. Xiang, A. Yagil, A. Barzdukas, L. Brennan, C. Campagnari, J. Incandela, J. Kim, A. J. Li, P. Masterson, H. Mei, J. Richman, U. Sarica, R. Schmitz, F. Setti, J. Sheplock, D. Stuart, T. Á. Vámi, S. Wang, A. Bornheim, O. Cerri, A. Latorre, J. Mao, H. B. Newman, G. Reales Gutiérrez, M. Spiropulu, J. R. Vlimant, C. Wang, S. Xie, R. Y. Zhu, J. Alison, S. An, M. B. Andrews, P. Bryant, M. Cremonesi, V. Dutta, T. Ferguson, A. Harilal, C. Liu, T. Mudholkar, S. Murthy, P. Palit, M. Paulini, A. Roberts, A. Sanchez, W. Terrill, J. P. Cumalat, W. T. Ford, A. Hart, A. Hassani, G. Karathanasis, N. Manganelli, A. Perloff, C. Savard, N. Schonbeck, K. Stenson, K. A. Ulmer, S. R. Wagner, N. Zipper, D. Zuolo, J. Alexander, S. Bright-Thonney, X. Chen, D. J. Cranshaw, J. Fan, X. Fan, S. Hogan, P. Kotamnives, J. Monroy, M. Oshiro, J. R. Patterson, J. Reichert, M. Reid, A. Ryd, J. Thom, P. Wittich, R. Zou, M. Albrow, M. Alyari, O. Amram, G. Apollinari, A. Apresyan, L. A. T. Bauerdick, D. Berry, J. Berryhill, P. C. Bhat, K. Burkett, J. N. Butler, A. Canepa, G. B. Cerati, H. W. K. Cheung, F. Chlebana, G. Cummings, J. Dickinson, I. Dutta, V. D. Elvira, Y. Feng, J. Freeman, A. Gandrakota, Z. Gecse, L. Gray, D. Green, A. Grummer, S. Grünendahl, D. Guerrero, O. Gutsche, R. M. Harris, R. Heller, T. C. Herwig, J. Hirschauer, L. Horyn, B. Jayatilaka, S. Jindariani, M. Johnson, U. Joshi, T. Klijnsma, B. Klima, K. H. M. Kwok, S. Lammel, D. Lincoln, R. Lipton, T. Liu, C. Madrid, K. Maeshima, C. Mantilla, D. Mason, P. McBride, P. Merkel, S. Mrenna, S. Nahn, J. Ngadiuba, D. Noonan, V. Papadimitriou, N. Pastika, K. Pedro, C. Pena, F. Ravera, A. Reinsvold Hall, L. Ristori, E. Sexton-Kennedy, N. Smith, A. Soha, L. Spiegel, S. Stoynev, J. Strait, L. Taylor, S. Tkaczyk, N. V. Tran, L. Uplegger, E. W. Vaandering, A. Whitbeck, I. Zoi, C. Aruta, P. Avery, D. Bourilkov, L. Cadamuro, P. Chang, V. Cherepanov, R. D. Field, E. Koenig, M. Kolosova, J. Konigsberg, A. Korytov, K. Matchev, N. Menendez, G. Mitselmakher, K. Mohrman, A. Muthirakalayil Madhu, N. Rawal, D. Rosenzweig, S. Rosenzweig, J. Wang, T. Adams, A. Al Kadhim, A. Askew, S. Bower, R. Habibullah, V. Hagopian, R. Hashmi, R. S. Kim, S. Kim, T. Kolberg, G. Martinez, H. Prosper, P. R. Prova, M. Wulansatiti, R. Yohay, J. Zhang, B. Alsufyani, M. M. Baarmand, S. Butalla, S. Das, T. Elkafrawy, M. Hohlmann, R. Kumar Verma, M. Rahmani, E. Yanes, M. R. Adams, A. Baty, C. Bennett, R. Cavanaugh, R. Escobar Franco, O. Evdokimov, C. E. Gerber, M. Hawksworth, A. Hingrajiya, D. J. Hofman, J. h. Lee, D. S. Lemos, A. H. Merrit, C. Mills, S. Nanda, G. Oh, B. Ozek, D. Pilipovic, R. Pradhan, E. Prifti, T. Roy, S. Rudrabhatla, M. B. Tonjes, N. Varelas, M. A. Wadud, Z. Ye, J. Yoo, M. Alhusseini, D. Blend, K. Dilsiz, L. Emediato, G. Karaman, O. K. Köseyan, J. -P. Merlo, A. Mestvirishvili, J. Nachtman, O. Neogi, H. Ogul, Y. Onel, A. Penzo, C. Snyder, E. Tiras, B. Blumenfeld, L. Corcodilos, J. Davis, A. V. Gritsan, L. Kang, S. Kyriacou, P. Maksimovic, M. Roguljic, J. Roskes, S. Sekhar, M. Swartz, A. Abreu, L. F. Alcerro Alcerro, J. Anguiano, P. Baringer, A. Bean, Z. Flowers, D. Grove, J. King, G. Krintiras, M. Lazarovits, C. Le Mahieu, J. Marquez, N. Minafra, M. Murray, M. Nickel, M. Pitt, S. Popescu, C. Rogan, C. Royon, R. Salvatico, S. Sanders, C. Smith, Q. Wang, G. Wilson, B. Allmond, R. Gujju Gurunadha, A. Ivanov, K. Kaadze, A. Kalogeropoulos, Y. Maravin, J. Natoli, D. Roy, G. Sorrentino, F. Rebassoo, D. Wright, A. Baden, A. Belloni, Y. M. Chen, S. C. Eno, N. J. Hadley, S. Jabeen, R. G. Kellogg, T. Koeth, Y. Lai, S. Lascio, A. C. Mignerey, S. Nabili, C. Palmer, C. Papageorgakis, M. M. Paranjpe, L. Wang, J. Bendavid, I. A. Cali, M. D’Alfonso, J. Eysermans, C. Freer, G. Gomez-Ceballos, M. Goncharov, G. Grosso, P. Harris, D. Hoang, D. Kovalskyi, J. Krupa, L. Lavezzo, Y. -J. Lee, K. Long, A. Novak, C. Paus, D. Rankin, C. Roland, G. Roland, S. Rothman, G. S. F. Stephans, Z. Wang, B. Wyslouch, T. J. Yang, B. Crossman, B. M. Joshi, C. Kapsiak, M. Krohn, D. Mahon, J. Mans, B. Marzocchi, S. Pandey, M. Revering, R. Rusack, R. Saradhy, N. Schroeder, N. Strobbe, L. M. Cremaldi, K. Bloom, D. R. Claes, G. Haza, J. Hossain, C. Joo, I. Kravchenko, J. E. Siado, W. Tabb, A. Vagnerini, A. Wightman, F. Yan, D. Yu, H. Bandyopadhyay, L. Hay, I. Iashvili, A. Kharchilava, M. Morris, D. Nguyen, S. Rappoccio, H. Rejeb Sfar, A. Williams, G. Alverson, E. Barberis, J. Dervan, Y. Haddad, Y. Han, A. Krishna, J. Li, M. Lu, G. Madigan, R. Mccarthy, D. M. Morse, V. Nguyen, T. Orimoto, A. Parker, L. Skinnari, D. Wood, J. Bueghly, Z. Chen, S. Dittmer, K. A. Hahn, Y. Liu, Y. Miao, D. G. Monk, M. H. Schmitt, A. Taliercio, M. Velasco, G. Agarwal, R. Band, R. Bucci, S. Castells, A. Das, R. Goldouzian, M. Hildreth, K. W. Ho, K. Hurtado Anampa, T. Ivanov, C. Jessop, K. Lannon, J. Lawrence, N. Loukas, L. Lutton, J. Mariano, N. Marinelli, I. Mcalister, T. McCauley, C. Mcgrady, C. Moore, Y. Musienko, H. Nelson, M. Osherson, A. Piccinelli, R. Ruchti, A. Townsend, Y. Wan, M. Wayne, H. Yockey, M. Zarucki, L. Zygala, A. Basnet, B. Bylsma, M. Carrigan, L. S. Durkin, C. Hill, M. Joyce, M. Nunez Ornelas, K. Wei, B. L. Winer, B. R. Yates, F. M. Addesa, H. Bouchamaoui, P. Das, G. Dezoort, P. Elmer, A. Frankenthal, B. Greenberg, N. Haubrich, G. Kopp, S. Kwan, D. Lange, A. Loeliger, D. Marlow, I. Ojalvo, J. Olsen, A. Shevelev, D. Stickland, C. Tully, S. Malik, A. S. Bakshi, V. E. Barnes, S. Chandra, R. Chawla, A. Gu, L. Gutay, M. Jones, A. W. Jung, D. Kondratyev, A. M. Koshy, M. Liu, G. Negro, N. Neumeister, G. Paspalaki, S. Piperov, V. Scheurer, J. F. Schulte, M. Stojanovic, J. Thieman, A. K. Virdi, F. Wang, W. Xie, J. Dolen, N. Parashar, A. Pathak, D. Acosta, T. Carnahan, K. M. Ecklund, P. J. Fernández Manteca, S. Freed, P. Gardner, F. J. M. Geurts, W. Li, O. Miguel Colin, B. P. Padley, R. Redjimi, J. Rotter, E. Yigitbasi, Y. Zhang, A. Bodek, P. de Barbaro, R. Demina, J. L. Dulemba, A. Garcia-Bellido, O. Hindrichs, A. Khukhunaishvili, N. Parmar, P. Parygin, E. Popova, R. Taus, K. Goulianos, B. Chiarito, J. P. Chou, S. V. Clark, D. Gadkari, Y. Gershtein, E. Halkiadakis, M. Heindl, C. Houghton, D. Jaroslawski, O. Karacheban, I. Laflotte, A. Lath, R. Montalvo, K. Nash, H. Routray, P. Saha, S. Salur, S. Schnetzer, S. Somalwar, R. Stone, S. A. Thayil, S. Thomas, J. Vora, H. Wang, H. Acharya, D. Ally, A. G. Delannoy, S. Fiorendi, S. Higginbotham, T. Holmes, A. R. Kanuganti, N. Karunarathna, L. Lee, E. Nibigira, S. Spanier, D. Aebi, M. Ahmad, O. Bouhali, R. Eusebi, J. Gilmore, T. Huang, T. Kamon, H. Kim, S. Luo, R. Mueller, D. Overton, D. Rathjens, A. Safonov, N. Akchurin, J. Damgov, V. Hegde, A. Hussain, Y. Kazhykarim, K. Lamichhane, S. W. Lee, A. Mankel, T. Peltola, I. Volobouev, E. Appelt, Y. Chen, S. Greene, A. Gurrola, W. Johns, R. Kunnawalkam Elayavalli, A. Melo, F. Romeo, P. Sheldon, S. Tuo, J. Velkovska, J. Viinikainen, B. Cardwell, B. Cox, J. Hakala, R. Hirosky, A. Ledovskoy, C. Neu, C. E. Perez Lara, S. Bhattacharya, P. E. Karchin, A. Aravind, S. Banerjee, K. Black, T. Bose, S. Dasu, I. De Bruyn, P. Everaerts, C. Galloni, H. He, M. Herndon, A. Herve, C. K. Koraka, A. Lanaro, R. Loveless, J. Madhusudanan Sreekala, A. Mallampalli, A. Mohammadi, S. Mondal, G. Parida, L. Pétré, D. Pinna, A. Savin, V. Shang, V. Sharma, W. H. Smith, D. Teague, H. F. Tsoi, W. Vetens, A. Warden, S. Afanasiev, V. Andreev, Yu. Andreev, T. Aushev, M. Azarkin, I. Azhgirey, A. Babaev, A. Belyaev, V. Blinov, E. Boos, V. Borshch, D. Budkouski, V. Bunichev, M. Chadeeva, V. Chekhovsky, R. Chistov, A. Dermenev, T. Dimova, D. Druzhkin, M. Dubinin, L. Dudko, A. Ershov, G. Gavrilov, V. Gavrilov, S. Gninenko, V. Golovtcov, N. Golubev, I. Golutvin, I. Gorbunov, Y. Ivanov, V. Kachanov, V. Karjavine, A. Karneyeu, V. Kim, M. Kirakosyan, D. Kirpichnikov, M. Kirsanov, V. Klyukhin, O. Kodolova, D. Konstantinov, V. Korenkov, A. Kozyrev, N. Krasnikov, A. Lanev, P. Levchenko, N. Lychkovskaya, V. Makarenko, A. Malakhov, V. Matveev, V. Murzin, A. Nikitenko, S. Obraztsov, V. Oreshkin, V. Palichik, V. Perelygin, M. Perfilov, S. Petrushanko, S. Polikarpov, V. Popov, O. Radchenko, R. Ryutin, M. Savina, V. Savrin, V. Shalaev, S. Shmatov, S. Shulha, Y. Skovpen, S. Slabospitskii, V. Smirnov, D. Sosnov, V. Sulimov, E. Tcherniaev, A. Terkulov, O. Teryaev, I. Tlisova, A. Toropin, L. Uvarov, A. Uzunian, A. Vorobyev, G. Vorotnikov, N. Voytishin, B. S. Yuldashev, A. Zarubin, I. Zhizhin, A. Zhokin

**Affiliations:** 1 Yerevan Physics Institute, Yerevan, Armenia; 2 Institut für Hochenergiephysik, Vienna, Austria; 3 Universiteit Antwerpen, Antwerp, Belgium; 4 Vrije Universiteit Brussel, Brussel, Belgium; 5 Université Libre de Bruxelles, Brussels, Belgium; 6 Ghent University, Ghent, Belgium; 7 Université Catholique de Louvain, Louvain-la-Neuve, Belgium; 8 Centro Brasileiro de Pesquisas Fisicas, Rio de Janeiro, Brazil; 9 Universidade do Estado do Rio de Janeiro, Rio de Janeiro, Brazil; 10 Universidade Estadual Paulista, Universidade Federal do ABC, São Paulo, Brazil; 11 Institute for Nuclear Research and Nuclear Energy, Bulgarian Academy of Sciences, Sofia, Bulgaria; 12 University of Sofia, Sofia, Bulgaria; 13 Instituto De Alta Investigación, Universidad de Tarapacá, Casilla 7 D, Arica, Chile; 14 Beihang University, Beijing, China; 15 Department of Physics, Tsinghua University, Beijing, China; 16 Institute of High Energy Physics, Beijing, China; 17 State Key Laboratory of Nuclear Physics and Technology, Peking University, Beijing, China; 18 Sun Yat-Sen University, Guangzhou, China; 19 University of Science and Technology of China, Hefei, China; 20 Nanjing Normal University, Nanjing, China; 21 Institute of Modern Physics and Key Laboratory of Nuclear Physics and Ion-beam Application (MOE), Fudan University, Shanghai, China; 22 Zhejiang University, Zhejiang, Hangzhou China; 23 Universidad de Los Andes, Bogota, Colombia; 24 Universidad de Antioquia, Medellin, Colombia; 25 University of Split, Faculty of Electrical Engineering, Mechanical Engineering and Naval Architecture, Split, Croatia; 26 University of Split, Faculty of Science, Split, Croatia; 27 Institute Rudjer Boskovic, Zagreb, Croatia; 28 University of Cyprus, Nicosia, Cyprus; 29 Charles University, Prague, Czech Republic; 30 Escuela Politecnica Nacional, Quito, Ecuador; 31 Universidad San Francisco de Quito, Quito, Ecuador; 32 Academy of Scientific Research and Technology of the Arab Republic of Egypt, Egyptian Network of High Energy Physics, Cairo, Egypt; 33 Center for High Energy Physics (CHEP-FU), Fayoum University, El-Fayoum, Egypt; 34 National Institute of Chemical Physics and Biophysics, Tallinn, Estonia; 35 Department of Physics, University of Helsinki, Helsinki, Finland; 36 Helsinki Institute of Physics, Helsinki, Finland; 37 Lappeenranta-Lahti University of Technology, Lappeenranta, Finland; 38 IRFU, CEA, Université Paris-Saclay, Gif-sur-Yvette, France; 39 Laboratoire Leprince-Ringuet, CNRS/IN2P3, Ecole Polytechnique, Institut Polytechnique de Paris, Palaiseau, France; 40 Université de Strasbourg, CNRS, IPHC UMR 7178, Strasbourg, France; 41 Institut de Physique des 2 Infinis de Lyon (IP2I ), Villeurbanne, France; 42 Georgian Technical University, Tbilisi, Georgia; 43 I. Physikalisches Institut, RWTH Aachen University, Aachen, Germany; 44 III. Physikalisches Institut A, RWTH Aachen University, Aachen, Germany; 45 III. Physikalisches Institut B, RWTH Aachen University, Aachen, Germany; 46 Deutsches Elektronen-Synchrotron, Hamburg, Germany; 47 University of Hamburg, Hamburg, Germany; 48 Karlsruher Institut fuer Technologie, Karlsruhe, Germany; 49 Institute of Nuclear and Particle Physics (INPP), NCSR Demokritos, Aghia Paraskevi, Greece; 50 National and Kapodistrian University of Athens, Athens, Greece; 51 National Technical University of Athens, Athens, Greece; 52 University of Ioánnina, Ioánnina, Greece; 53 HUN-REN Wigner Research Centre for Physics, Budapest, Hungary; 54 MTA-ELTE Lendület CMS Particle and Nuclear Physics Group, Eötvös Loránd University, Budapest, Hungary; 55 Faculty of Informatics, University of Debrecen, Debrecen, Hungary; 56 Institute of Nuclear Research ATOMKI, Debrecen, Hungary; 57 Karoly Robert Campus, MATE Institute of Technology, Gyongyos, Hungary; 58 Panjab University, Chandigarh, India; 59 University of Delhi, Delhi, India; 60 Saha Institute of Nuclear Physics, HBNI, Kolkata, India; 61 Indian Institute of Technology Madras, Madras, India; 62 Tata Institute of Fundamental Research-A, Mumbai, India; 63 Tata Institute of Fundamental Research-B, Mumbai, India; 64 National Institute of Science Education and Research, An OCC of Homi Bhabha National Institute, Bhubaneswar, Odisha India; 65 Indian Institute of Science Education and Research (IISER), Pune, India; 66 Isfahan University of Technology, Isfahan, Iran; 67 Institute for Research in Fundamental Sciences (IPM), Tehran, Iran; 68 University College Dublin, Dublin, Ireland; 69 INFN Sezione di Bari, Università di Bari, Politecnico di Bari, Bari, Italy; 70 INFN Sezione di Bologna, Università di Bologna, Bologna, Italy; 71 INFN Sezione di Catania, Università di Catania, Catania, Italy; 72 INFN Sezione di Firenze, Università di Firenze, Florence, Italy; 73 INFN Laboratori Nazionali di Frascati, Frascati, Italy; 74 INFN Sezione di Genova, Università di Genova, Genoa, Italy; 75 INFN Sezione di Milano-Bicocca, Università di Milano-Bicocca, Milan, Italy; 76 INFN Sezione di Napoli, Università di Napoli ‘Federico II’, Naples, Italy; Università della Basilicata, Potenza, Italy; Scuola Superiore Meridionale (SSM), Naples, Italy; 77 INFN Sezione di Padova, Università di Padova, Padua, Italy; Università di Trento, Trento, Italy; 78 INFN Sezione di Pavia, Università di Pavia, Pavia, Italy; 79 INFN Sezione di Perugia, Università di Perugia, Perugia, Italy; 80 INFN Sezione di Pisa, Università di Pisa, Scuola Normale Superiore di Pisa, Pisa, Italy; Università di Siena, Siena, Italy; 81 INFN Sezione di Roma, Sapienza Università di Roma, Rome, Italy; 82 INFN Sezione di Torino, Università di Torino, Turin, Italy; Università del Piemonte Orientale, Novara, Italy; 83 INFN Sezione di Trieste, Università di Trieste, Trieste, Italy; 84 Kyungpook National University, Daegu, Korea; 85 Department of Mathematics and Physics-GWNU, Gangneung, Korea; 86 Chonnam National University, Institute for Universe and Elementary Particles, Kwangju, Korea; 87 Hanyang University, Seoul, Korea; 88 Korea University, Seoul, Korea; 89 Department of Physics, Kyung Hee University, Seoul, Korea; 90 Sejong University, Seoul, Korea; 91 Seoul National University, Seoul, Korea; 92 University of Seoul, Seoul, Korea; 93 Department of Physics, Yonsei University, Seoul, Korea; 94 Sungkyunkwan University, Suwon, Korea; 95 College of Engineering and Technology, American University of the Middle East (AUM), Dasman, Kuwait; 96 Riga Technical University, Riga, Latvia; 97 University of Latvia (LU), Riga, Latvia; 98 Vilnius University, Vilnius, Lithuania; 99 National Centre for Particle Physics, Universiti Malaya, Kuala Lumpur, Malaysia; 100 Universidad de Sonora (UNISON), Hermosillo, Mexico; 101 Centro de Investigacion y de Estudios Avanzados del IPN, Mexico City, Mexico; 102 Universidad Iberoamericana, Mexico City, Mexico; 103 Benemerita Universidad Autonoma de Puebla, Puebla, Mexico; 104 University of Montenegro, Podgorica, Montenegro; 105 University of Canterbury, Christchurch, New Zealand; 106 National Centre for Physics, Quaid-I-Azam University, Islamabad, Pakistan; 107 AGH University of Krakow, Faculty of Computer Science, Electronics and Telecommunications, Krakow, Poland; 108 National Centre for Nuclear Research, Swierk, Poland; 109 Institute of Experimental Physics, Faculty of Physics, University of Warsaw, Warsaw, Poland; 110 Warsaw University of Technology, Warsaw, Poland; 111 Laboratório de Instrumentação e Física Experimental de Partículas, Lisbon, Portugal; 112 Faculty of Physics, University of Belgrade, Belgrade, Serbia; 113 VINCA Institute of Nuclear Sciences, University of Belgrade, Belgrade, Serbia; 114 Centro de Investigaciones Energéticas Medioambientales y Tecnológicas (CIEMAT), Madrid, Spain; 115 Universidad Autónoma de Madrid, Madrid, Spain; 116 Instituto Universitario de Ciencias y Tecnologías Espaciales de Asturias (ICTEA), Universidad de Oviedo, Oviedo, Spain; 117 Instituto de Física de Cantabria (IFCA), CSIC-Universidad de Cantabria, Santander, Spain; 118 University of Colombo, Colombo, Sri Lanka; 119 Department of Physics, University of Ruhuna, Matara, Sri Lanka; 120 CERN, European Organization for Nuclear Research, Geneva, Switzerland; 121 Paul Scherrer Institut, Villigen, Switzerland; 122 ETH Zurich-Institute for Particle Physics and Astrophysics (IPA), Zurich, Switzerland; 123 Universität Zürich, Zurich, Switzerland; 124 National Central University, Chung-Li, Taiwan; 125 National Taiwan University (NTU), Taipei, Taiwan; 126 High Energy Physics Research Unit, Department of Physics, Faculty of Science, Chulalongkorn University, Bangkok, Thailand; 127 Physics Department, Science and Art Faculty, Çukurova University, Adana, Turkey; 128 Physics Department, Middle East Technical University, Ankara, Turkey; 129 Bogazici University, Istanbul, Turkey; 130 Istanbul Technical University, Istanbul, Turkey; 131 Istanbul University, Istanbul, Turkey; 132 Yildiz Technical University, Istanbul, Turkey; 133 Institute for Scintillation Materials of National Academy of Science of Ukraine, Kharkiv, Ukraine; 134 National Science Centre, Kharkiv Institute of Physics and Technology, Kharkiv, Ukraine; 135 University of Bristol, Bristol, UK; 136 Rutherford Appleton Laboratory, Didcot, UK; 137 Imperial College, London, UK; 138 Brunel University, Uxbridge, UK; 139 Baylor University, Waco, TX USA; 140 Catholic University of America, Washington, DC USA; 141 The University of Alabama, Tuscaloosa, AL USA; 142 Boston University, Boston, MA USA; 143 Brown University, Providence, RI USA; 144 University of California, Davis, CA USA; 145 University of California, Los Angeles, CA USA; 146 University of California, Riverside, Riverside, CA USA; 147 University of California, San Diego, La Jolla, CA USA; 148 Department of Physics, University of California, Santa Barbara, Santa Barbara, CA USA; 149 California Institute of Technology, Pasadena, CA USA; 150 Carnegie Mellon University, Pittsburgh, PA USA; 151 University of Colorado Boulder, Boulder, CO USA; 152 Cornell University, Ithaca, NY USA; 153 Fermi National Accelerator Laboratory, Batavia, IL USA; 154 University of Florida, Gainesville, FL USA; 155 Florida State University, Tallahassee, FL USA; 156 Florida Institute of Technology, Melbourne, FL USA; 157 University of Illinois Chicago, Chicago, USA; 158 The University of Iowa, Iowa City, IA USA; 159 Johns Hopkins University, Baltimore, MD USA; 160 The University of Kansas, Lawrence, KS USA; 161 Kansas State University, Manhattan, KS USA; 162 Lawrence Livermore National Laboratory, Livermore, CA USA; 163 University of Maryland, College Park, MD USA; 164 Massachusetts Institute of Technology, Cambridge, MA USA; 165 University of Minnesota, Minneapolis, MN USA; 166 University of Mississippi, Oxford, MS USA; 167 University of Nebraska-Lincoln, Lincoln, NE USA; 168 State University of New York at Buffalo, Buffalo, NY USA; 169 Northeastern University, Boston, MA USA; 170 Northwestern University, Evanston, IL USA; 171 University of Notre Dame, Notre Dame, IN USA; 172 The Ohio State University, Columbus, OH USA; 173 Princeton University, Princeton, NJ USA; 174 University of Puerto Rico, Mayaguez, PR USA; 175 Purdue University, Indiana, WL USA; 176 Purdue University Northwest, Hammond, IN USA; 177 Rice University, Houston, TX USA; 178 University of Rochester, Rochester, NY USA; 179 The Rockefeller University, New York, NY USA; 180 Rutgers, The State University of New Jersey, Piscataway, NJ USA; 181 University of Tennessee, Knoxville, TN USA; 182 Texas A &M University, College Station, TX USA; 183 Texas Tech University, Lubbock, TX USA; 184 Vanderbilt University, Nashville, TN USA; 185 University of Virginia, Charlottesville, VA USA; 186 Wayne State University, Detroit, MI USA; 187 University of Wisconsin-Madison, Madison, WI USA; 188 Authors Affiliated with an Institute or an International Laboratory Covered by a Cooperation Agreement with CERN, Geneva, Switzerland; 189 Yerevan State University, Yerevan, Armenia; 190 TU Wien, Vienna, Austria; 191 Institute of Basic and Applied Sciences, Faculty of Engineering, Arab Academy for Science, Technology and Maritime Transport, Alexandria, Egypt; 192 Ghent University, Ghent, Belgium; 193 Universidade Estadual de Campinas, Campinas, Brazil; 194 Federal University of Rio Grande do Sul, Porto Alegre, Brazil; 195 UFMS, Nova Andradina, Brazil; 196 Nanjing Normal University, Nanjing, China; 197 The University of Iowa, Iowa City, IA USA; 198 University of Chinese Academy of Sciences, Beijing, China; 199 China Center of Advanced Science and Technology, Beijing, China; 200 University of Chinese Academy of Sciences, Beijing, China; 201 China Spallation Neutron Source, Guangdong, China; 202 Henan Normal University, Xinxiang, China; 203 Université Libre de Bruxelles, Brussels, Belgium; 204 an Institute or an International Laboratory Covered by a Cooperation Agreement with CERN, Geneva, Switzerland; 205 Cairo University, Cairo, Egypt; 206 Suez University, Suez, Egypt; 207 British University in Egypt, Cairo, Egypt; 208 Purdue University, West Lafayette, IN USA; 209 Université de Haute Alsace, Mulhouse, France; 210 Department of Physics, Tsinghua University, Beijing, China; 211 The University of the State of Amazonas, Manaus, Brazil; 212 Erzincan Binali Yildirim University, Erzincan, Turkey; 213 University of Hamburg, Hamburg, Germany; 214 III. Physikalisches Institut A, RWTH Aachen University, Aachen, Germany; 215 Isfahan University of Technology, Isfahan, Iran; 216 Bergische University Wuppertal (BUW), Wuppertal, Germany; 217 Brandenburg University of Technology, Cottbus, Germany; 218 Forschungszentrum Jülich, Juelich, Germany; 219 CERN, European Organization for Nuclear Research, Geneva, Switzerland; 220 Institute of Physics, University of Debrecen, Debrecen, Hungary; 221 Institute of Nuclear Research ATOMKI, Debrecen, Hungary; 222 Universitatea Babes-Bolyai-Facultatea de Fizica, Cluj-Napoca, Romania; 223 MTA-ELTE Lendület CMS Particle and Nuclear Physics Group, Eötvös Loránd University, Budapest, Hungary; 224 Physics Department, Faculty of Science, Assiut University, Assiut, Egypt; 225 HUN-REN Wigner Research Centre for Physics, Budapest, Hungary; 226 Punjab Agricultural University, Ludhiana, India; 227 University of Visva-Bharati, Santiniketan, India; 228 Indian Institute of Science (IISc), Bangalore, India; 229 Birla Institute of Technology, Mesra, Mesra, India; 230 IIT Bhubaneswar, Bhubaneswar, India; 231 Institute of Physics, Bhubaneswar, India; 232 University of Hyderabad, Hyderabad, India; 233 Deutsches Elektronen-Synchrotron, Hamburg, Germany; 234 Department of Physics, Isfahan University of Technology, Isfahan, Iran; 235 Sharif University of Technology, Tehran, Iran; 236 Department of Physics, University of Science and Technology of Mazandaran, Behshahr, Iran; 237 Helwan University, Cairo, Egypt; 238 Italian National Agency for New Technologies, Energy and Sustainable Economic Development, Bologna, Italy; 239 Centro Siciliano di Fisica Nucleare e di Struttura Della Materia, Catania, Italy; 240 Università degli Studi Guglielmo Marconi, Rome, Italy; 241 Scuola Superiore Meridionale, Università di Napoli ‘Federico II’, Naples, Italy; 242 Fermi National Accelerator Laboratory, Batavia, IL USA; 243 Ain Shams University, Cairo, Egypt; 244 Consiglio Nazionale delle Ricerche-Istituto Officina dei Materiali, Perugia, Italy; 245 Department of Applied Physics, Faculty of Science and Technology, Universiti Kebangsaan Malaysia, Bangi, Malaysia; 246 Consejo Nacional de Ciencia y Tecnología, Mexico City, Mexico; 247 Trincomalee Campus, Eastern University, Nilaveli, Sri Lanka; 248 Saegis Campus, Nugegoda, Sri Lanka; 249 National and Kapodistrian University of Athens, Athens, Greece; 250 Ecole Polytechnique Fédérale Lausanne, Lausanne, Switzerland; 251 Universität Zürich, Zurich, Switzerland; 252 Stefan Meyer Institute for Subatomic Physics, Vienna, Austria; 253 Laboratoire d’Annecy-le-Vieux de Physique des Particules, IN2P3-CNRS, Annecy-le-Vieux, France; 254 Research Center of Experimental Health Science, Near East University, Mersin, Turkey; 255 Konya Technical University, Konya, Turkey; 256 Izmir Bakircay University, Izmir, Turkey; 257 Adiyaman University, Adiyaman, Turkey; 258 Bozok Universitetesi Rektörlügü, Yozgat, Turkey; 259 Marmara University, Istanbul, Turkey; 260 Milli Savunma University, Istanbul, Turkey; 261 Kafkas University, Kars, Turkey; 262 Istanbul Okan University, Istanbul, Turkey; 263 Hacettepe University, Ankara, Turkey; 264 Faculty of Engineering, Istanbul University-Cerrahpasa, Istanbul, Turkey; 265 Yildiz Technical University, Istanbul, Turkey; 266 Vrije Universiteit Brussel, Brussels, Belgium; 267 School of Physics and Astronomy, University of Southampton, Southampton, UK; 268 IPPP Durham University, Durham, UK; 269 Faculty of Science, Monash University, Clayton, Australia; 270 Università di Torino, Turin, Italy; 271 Bethel University, St. Paul, MN USA; 272 Karamanoğlu Mehmetbey University, Karaman, Turkey; 273 California Institute of Technology, Pasadena, CA USA; 274 United States Naval Academy, Annapolis, MD USA; 275 Bingol University, Bingol, Turkey; 276 Georgian Technical University, Tbilisi, Georgia; 277 Sinop University, Sinop, Turkey; 278 Erciyes University, Kayseri, Turkey; 279 Horia Hulubei National Institute of Physics and Nuclear Engineering (IFIN-HH), Bucharest, Romania; 280 an Institute or an International Laboratory Covered by a Cooperation Agreement with CERN, Geneva, Switzerland; 281 Texas A &M University at Qatar, Doha, Qatar; 282 Kyungpook National University, Daegu, Korea; 283 Another Institute or International Laboratory Covered by a Cooperation Agreement with CERN, Geneva, Switzerland; 284 Universiteit Antwerpen, Antwerp, Belgium; 285 Yerevan Physics Institute, Yerevan, Armenia; 286 Northeastern University, Boston, MA USA; 287 Imperial College, London, UK; 288 Institute of Nuclear Physics of the Uzbekistan Academy of Sciences, Tashkent, Uzbekistan; 289 CERN, 1211 Geneva 23, Switzerland

## Abstract

A search for ${\text {Z}{}{}} {\text {Z}{}{}}$ and ${\text {Z}{}{}} {\text {H}{}{}}$ production in the ${\text {b}{}{}} {\bar{{\text {b}{}{}}}{}{}} {\text {b}{}{}} {\bar{{\text {b}{}{}}}{}{}}$ final state is presented, where H is the standard model (SM) Higgs boson. The search uses an event sample of proton-proton collisions corresponding to an integrated luminosity of 133$\,\text {fb}^{-1}$ collected at a center-of-mass energy of 13$\,\text {Te}\hspace{-.08em}\text {V}$ with the CMS detector at the CERN LHC. The analysis introduces several novel techniques for deriving and validating a multi-dimensional background model based on control samples in data. A multiclass multivariate classifier customized for the ${\text {b}{}{}} {\bar{{\text {b}{}{}}}{}{}} {\text {b}{}{}} {\bar{{\text {b}{}{}}}{}{}}$ final state is developed to derive the background model and extract the signal. The data are found to be consistent, within uncertainties, with the SM predictions. The observed (expected) upper limits at 95% confidence level are found to be 3.8 (3.8) and 5.0 (2.9) times the SM prediction for the ${\text {Z}{}{}} {\text {Z}{}{}}$ and ${\text {Z}{}{}} {\text {H}{}{}}$ production cross sections, respectively.

## Introduction

The observation of Higgs boson pair (${\text {H}{}{}} {\text {H}{}{}}$) production is an important goal of the High-Luminosity LHC (HL-LHC) program [1]. This process is sensitive to the self-coupling ($\lambda$) of the Higgs boson, a crucial parameter of the standard model (SM) that has not yet been measured [2]. Estimates of the sensitivity to the ${\text {H}{}{}} {\text {H}{}{}}$ processes indicate that the SM cross section is at the edge of what is observable with an integrated luminosity of 3000$\,\text {fb}^{-1}$ at the HL-LHC [3]. An observation of ${\text {H}{}{}} {\text {H}{}{}}$ production and a measurement of $\lambda$ will require the combination of the ${\text {b}{}{}} {\bar{{\text {b}{}{}}}{}{}} {{\upgamma }{}{}} {{\upgamma }{}{}}$, ${\text {b}{}{}} {\bar{{\text {b}{}{}}}{}{}} {{\uptau }{}{}} {{\uptau }{}{}}$, and ${\text {b}{}{}} {\bar{{\text {b}{}{}}}{}{}} {\text {b}{}{}} {\bar{{\text {b}{}{}}}{}{}}$ (4b) decay modes [4–10]. The 4b channel has the largest ${\text {H}{}{}} {\text {H}{}{}}$ decay branching fraction but suffers from a large background composed of jets produced through the strong interaction, referred to as quantum chromodynamic (QCD) multijet events. This background is challenging to model with simulation; the QCD multijet predictions lack sufficient accuracy and it is not possible to generate sufficiently large samples. Extracting all available information from the 4b decay mode will thus require the development and validation of a multi-dimensional background model based on control samples in data.

In previous ${\text {H}{}{}} {\text {H}{}{}}$ $\rightarrow$ 4b searches [4, 9, 11–15], the QCD multijet background is determined in a signal-free control region using a variant of the “ABCD” or matrix method [16–18]. The background prediction in the signal region (SR) requires an extrapolation to a different region of phase space. This extrapolation is a significant source of systematic uncertainty that limits the ultimate sensitivity of the analysis. A common approach for assessing this extrapolation uncertainty is to validate the background prediction in a third, statistically independent validation region (VR) [4, 9, 11–15] that is dominated by background. This strategy can address how accurately the background model extrapolates to a different region of phase space, but does not directly test the extrapolation into the SR. In addition, it inevitably suffers from a lack of statistical power in the phase space with the highest signal-to-background ratio; the selection that makes the VR background-dominated depletes the most sensitive phase space of the SR.

This paper introduces a new method to overcome these limitations by validating the background model with data samples obtained using hemisphere mixing [19, 20], referred to as synthetic data samples. These synthetic data samples allow for the validation of the extrapolation of the background model to the relevant SR and avoid the problem of low statistical power in the most signal-like phase space. This technique also provides a way to determine the expected variance of the background prediction, resulting from the finite size of the data sample used to fit the model.

The ${\text {Z}{}{}} {\text {Z}{}{}} \rightarrow 4{\text {b}{}{}}$ and ${\text {Z}{}{}} {\text {H}{}{}} \rightarrow 4{\text {b}{}{}}$ processes share the final state and all the experimental challenges of the ${\text {H}{}{}} {\text {H}{}{}} \rightarrow 4{\text {b}{}{}}$ analysis, but have production cross sections that are expected to be 31 (${\text {Z}{}{}} {\text {Z}{}{}}$) [21] and 8 (${\text {Z}{}{}} {\text {H}{}{}}$) [22] times larger than ${\text {H}{}{}} {\text {H}{}{}}$. In addition, the ${\text {Z}{}{}} {\text {Z}{}{}}$ and ${\text {Z}{}{}} {\text {H}{}{}}$ processes are well established experimentally, both having been observed and measured using channels in which the Z decays to leptons [23–27].

This paper presents a search for ${\text {Z}{}{}} {\text {Z}{}{}} \rightarrow$ 4b and ${\text {Z}{}{}} {\text {H}{}{}} \rightarrow$ 4b production using 133$\,\text {fb}^{-1}$ of proton-proton (${\text {p}{}{}} {\text {p}{}{}}$) collisions at $\sqrt{s} = 13\,\text {Te}\hspace{-.08em}\text {V}$, collected by the CMS experiment at the LHC. The analysis introduces several new techniques for deriving and validating the background model. A multiclass multivariate classifier, which uses convolutional layers to solve the combinatoric jet-pairing problem, has been designed with an architecture customized to the 4b final state. The classifier is used both for signal-versus-background discrimination as well as for the derivation and validation of the background model. While these techniques are developed and demonstrated in the ${\text {Z}{}{}} {\text {Z}{}{}}$ and ${\text {Z}{}{}} {\text {H}{}{}}$ $\rightarrow$ 4b searches, they are directly applicable to the ${\text {H}{}{}} {\text {H}{}{}} \rightarrow 4{\text {b}{}{}}$ analysis and the measurement of $\lambda$.

The remainder of the paper is organized as follows. The CMS detector and the reconstruction and identification of physics objects used in this analysis are described in Sect. 2. Section 3 discusses the data and the simulated events used. Details of the event selection are presented in Sect. 4. Section 5 describes the architecture of the multivariate classifier used throughout the analysis. The background modeling method is described in Sect. 6 and its validation in Sect. 7. The construction of the synthetic data samples are described in Sect. 7.1, and the evaluation of the background uncertainties in Sect. 7.2. Other sources of systematic uncertainty are detailed in Sect. 8. Finally, the results are reported in Sect. 9 and a summary is provided in Sect. 10. The tabulated results are provided in a HEPData record [28].

## The CMS detector

The CMS apparatus [29, 30] is a multipurpose, nearly hermetic detector, designed to trigger on [31, 32] and identify electrons, muons, photons, and (charged and neutral) hadrons [33–35]. The central feature of the CMS detector is a superconducting solenoid, providing a magnetic field of 3.8 T. Within the solenoid volume are a silicon pixel and strip tracker, a lead tungstate crystal electromagnetic calorimeter, and a brass and scintillator hadron calorimeter, each composed of a barrel and two endcap sections. Forward calorimeters extend the pseudorapidity ($\eta$) coverage provided by the barrel and endcap detectors. Muons are detected in gas-ionization chambers embedded in the steel flux-return yoke outside the solenoid. A more detailed description of the CMS detector, together with a definition of the coordinate system used and the relevant kinematic variables, can be found in Ref. [29].

Events of interest are selected using a two-tiered trigger system. The first level (L1), composed of custom hardware processors, uses information from the calorimeters and muon detectors to select events at a rate of around 100 kHz within a fixed latency of 4$\,\mu \text {s}$ [31]. The second level, known as the high-level trigger (HLT), consists of a farm of processors running a version of the full event reconstruction software optimized for fast processing, and reduces the event rate to around 1 kHz before data storage [32].

The event reconstruction is based on the particle-flow (PF) algorithm [36], which aims to reconstruct and identify each individual particle (PF candidate) in an event with an optimized combination of information from the various elements of the CMS detector. The PF candidates are classified as electrons, muons, photons, and charged or neutral hadrons. The primary vertex is taken to be the vertex corresponding to the hardest scattering in the event, evaluated using tracking information alone, as described in Section 9.4.1 of Ref. [37].

Jets are reconstructed from PF candidates, clustered using the anti-$k_{\textrm{T}}$ algorithm [38, 39] with a distance parameter of 0.4. The jet momentum is determined as the vectorial sum of all particle momenta in the jet, and is found from simulation to be, on average, within 5–10% of the true momentum over the whole $p_{\textrm{T}}$ spectrum and detector acceptance. Additional collisions within the same or nearby bunch crossings (pileup) can give rise to jets not coming from the hard-scattering process or contribute additional tracks and calorimetric energy depositions, increasing the apparent jet momentum. To mitigate this effect, tracks identified as originating from pileup vertices are discarded and an offset correction is applied to account for remaining contributions [40]. Jet energy corrections are derived from simulation studies so that the average measured energy of jets becomes identical to that of particle-level jets. In situ measurements of the momentum balance in dijets, $\gamma +\textrm{jets}$, ${\text {Z}{}{}} +\textrm{jets}$, and multijet events are used to determine any residual differences between the jet energy scale in data and in simulation, and appropriate corrections are applied [41, 42]. Jets originating from b quarks are identified using the DeepJet algorithm [43], a deep neural network combining secondary vertex properties, track-based variables, and PF jet constituents (neutral and charged particle candidates). The efficiency of b (light flavor and gluon) jets to pass the b-tagging requirement used in this analysis is data-set dependent and varies within the 50–60% (0.05–0.5%) range.

## Data and simulation

This analysis is performed on data collected during three years of data taking at $\sqrt{s}$ = 13$\,\text {Te}\hspace{-.08em}\text {V}$. The combined data set corresponds to an integrated luminosity of 133$\,\text {fb}^{-1}$ collected during 2016–2018 [44–46].

Events are selected at L1 using triggers requiring the presence of at least four jets in the tracker acceptance ($|\eta | < 2.5$) and large $H_{\textrm{T}}$, defined as the scalar sum of the $p_{\textrm{T}}$ of the reconstructed jets in the event. During the 2016 data taking, events are required to have either $H_{\textrm{T}} > 280\,\text {Ge}\hspace{-.08em}\text {V}$ or at least four jets with $p_{\textrm{T}} > 50\,\text {Ge}\hspace{-.08em}\text {V}$. In the 2017 data set, events are required to have $H_{\textrm{T}} > 280\,\text {Ge}\hspace{-.08em}\text {V}$ and the four leading jets are required to pass staggered $p_{\textrm{T}}$ thresholds of 70, 55, 40, and 35$\,\text {Ge}\hspace{-.08em}\text {V}$. In the 2018 data set, the $H_{\textrm{T}}$ requirement was raised to 320$\,\text {Ge}\hspace{-.08em}\text {V}$ and the lowest jet $p_{\textrm{T}}$ threshold was raised to 40$\,\text {Ge}\hspace{-.08em}\text {V}$.

Events are selected in the HLT using a combination of triggers requiring the presence of jets coming from the hadronization of b quarks (b jets). Events are required to have at least four jets, at least three of which are identified as arising from a bottom quark (${\text {b}{}{}}$ tagged). In the 2016 data set, events are required to have either at least four jets with transverse momentum $p_{\textrm{T}} > 45\,\text {Ge}\hspace{-.08em}\text {V}$, or two or more jets with $p_{\textrm{T}} > 90\,\text {Ge}\hspace{-.08em}\text {V}$ and two or more jets with $p_{\textrm{T}} > 30\,\text {Ge}\hspace{-.08em}\text {V}$. In the 2017 data set, an $H_{\textrm{T}}$ requirement of 300$\,\text {Ge}\hspace{-.08em}\text {V}$ was added to match the threshold at L1, and the four highest-$p_{\textrm{T}}$ jets were required to pass staggered $p_{\textrm{T}}$ thresholds of 75, 60, 45, and 40$\,\text {Ge}\hspace{-.08em}\text {V}$. The $H_{\textrm{T}}$ threshold was raised to 330$\,\text {Ge}\hspace{-.08em}\text {V}$ in 2018. The b tagging was performed in HLT using the CSV algorithm [47] in 2016–2017, and with the DeepCSV algorithm [43] in 2018. Following the selection described in Sect. 4, this combination of triggers has an approximate efficiency of 20% for simulated signals with di-boson invariant mass near 200$\,\text {Ge}\hspace{-.08em}\text {V}$, rising to 90% efficiency for masses greater than 600$\,\text {Ge}\hspace{-.08em}\text {V}$.

Simulated events are used to model ${\text {Z}{}{}} {\text {Z}{}{}}$, ${\text {Z}{}{}} {\text {H}{}{}}$, and ${\text {H}{}{}} {\text {H}{}{}}$ events and the background from top quark pair (${{\textrm{t}}{}{}} {}{\bar{\textrm{t}}{}{}}$) production. The ${{\textrm{t}}{}{}} {}{\bar{\textrm{t}}{}{}}$ process is generated at next-to-leading-order (NLO) accuracy in QCD [48] with powheg v2.0 [49, 50]. The dominant background from QCD multijet events is modeled using control samples in data.

Events from ${\text {Z}{}{}} {\text {Z}{}{}}$ production are generated with MadGraph 5_amc@nlo v2.4.2 [51] at NLO QCD with the FxFx merging scheme [52] and include up to two additional partons. The SM prediction for the total ${\text {Z}{}{}} {\text {Z}{}{}}$ production cross section in ${\text {p}{}{}} {\text {p}{}{}}$ collisions at $\sqrt{s} = 13\,\text {Te}\hspace{-.08em}\text {V}$ is $15.0^{+0.7}_{-0.6}$ pb, taken from Ref. [21].

The quark-induced ${\text {Z}{}{}} {\text {H}{}{}}$ signal process is generated at NLO accuracy [53] using the powheg v2 event generator extended with the MiNLO procedure [54, 55], while the gluon-induced ${\text {Z}{}{}} {\text {H}{}{}}$ process is simulated at LO accuracy with powheg v2. The SM prediction for the total ${\text {Z}{}{}} {\text {H}{}{}}$ production cross section, computed at next-to-next-to-leading-order accuracy in QCD, is $0.88 \pm 0.03$ pb for $m_{\textrm{H}} = 125\,\text {Ge}\hspace{-.08em}\text {V}$ [22]. The SM branching fractions of 58.2 and 15.1% are taken for H $\rightarrow$ bb and Z $\rightarrow$ bb decays, respectively [22, 56, 57], again assuming $m_{\textrm{H}} = 125\,\text {Ge}\hspace{-.08em}\text {V}$.

Events from ${\text {H}{}{}} {\text {H}{}{}}$ production used to train the classifier are simulated at NLO accuracy [58] with powheg v2. The dominant SM ${\text {H}{}{}} {\text {H}{}{}}$ production mode in ${\text {p}{}{}} {\text {p}{}{}}$ collisions at $\sqrt{s} = 13\,\text {Te}\hspace{-.08em}\text {V}$ is through the gluon-fusion mechanism; the predicted cross section, computed at next-to-next-to-leading-order accuracy, is $31.1^{+2.1}_{-7.2}$ fb [59–66].

For both signal and background events, multiple-parton interactions, parton shower, and hadronization effects are simulated with pythia v8.226 for 2016 and pythia v8.230 for 2017–2018 [67]. For 2016, the CP5 tune [68] is used for the ${{\textrm{t}}{}{}} {}{\bar{\textrm{t}}{}{}}$ samples and the CUETP8M1 tune [69] is used for all the others; for 2017–2018, the CP5 tune is used throughout all samples. The NNPDF 3.0 [70] (NNPDF 3.1 [71]) parton distribution functions (PDFs) are used to simulate the samples corresponding to the 2016 (2017–2018) data sets. For all simulated samples, the CMS detector response is modeled with Geant4 [72]. Pileup collisions are simulated and added to the hard-scattering process for all samples. The simulated events are weighted to match the distribution of reconstructed primary vertices observed in data.

## Event selection

Selected events must have at least four jets with $p_{\textrm{T}} > 40$ $\,\text {Ge}\hspace{-.08em}\text {V}$ and $|\eta | < 2.4$ that are b-tagged by the DeepJet algorithm. Events passing this selection are referred to as the four-tag sample. The four jets with the highest b tagging score are paired to form Z or H candidates (“boson-candidate jets” in what follows). A dedicated correction to the b jet energy scale, based on a regression technique that takes properties of the jets into account, is applied to the boson-candidate jets. This improves the determination of the jet momentum by up to 15% [73].

Initially, all three possible jet pairings are considered. To reduce the combinatorial background, pairs of jets with an invariant mass roughly consistent with the Z and H masses are retained. Jet pairings must satisfy

1 $$\begin{aligned} \begin{aligned} 52&< m_{\textrm{jj}}^{\text {lead}}< 180\,\text {Ge}\hspace{-.08em}\text {V} \quad \text {and} \\ 50&< m_{\textrm{jj}}^{\text {subl}}< 173\,\text {Ge}\hspace{-.08em}\text {V}, \end{aligned} \end{aligned}$$

where $m_{\textrm{jj}}^{\text {lead}}$ and $m_{\textrm{jj}}^{\text {subl}}$ are the invariant masses of the leading and subleading boson candidates, respectively. The leading boson candidate is defined as the one with the highest scalar sum of jet $p_{\textrm{T}}$. From simulation it is found that this sorting tends to bias the leading dijet mass distribution upwards by 2% and the subleading – downwards by 2%. This is taken into account when defining the dijet mass regions used in the analysis.

The angle between the decay products of the bosons in the laboratory frame provides another handle to reduce the background. This angle depends on the Lorentz boost of the bosons and, thus, on the four-jet invariant mass $m_{\textrm{4j}}$. The pairings of jets associated with the boson candidates satisfy the following requirements

2 $$\begin{aligned} \begin{aligned} \frac{360\,\text {Ge}\hspace{-.08em}\text {V}}{m_{\textrm{4j}}} - 0.5<&\Delta \text {R}_{\text {jj}}^{\text {lead}}< \max \bigg [1.5, \frac{650\,\text {Ge}\hspace{-.08em}\text {V}}{m_{\textrm{4j}}} + 0.5 \bigg ]\\ {\hbox {and}\hspace{0.0pt}}\\ \frac{235\,\text {Ge}\hspace{-.08em}\text {V}}{m_{\textrm{4j}}}<&\Delta \text {R}_{\text {jj}}^{\text {subl}}< \max \bigg [1.5, \frac{650\,\text {Ge}\hspace{-.08em}\text {V}}{m_{\textrm{4j}}} + 0.7 \bigg ], \end{aligned} \end{aligned}$$

where $\Delta \text {R}_{\text {jj}}^{\text {lead}}$ and $\Delta \text {R}_{\text {jj}}^{\text {subl}}$ are the angular separations between the jets in the leading and subleading boson candidates, respectively. The angular separation is defined as $\Delta R = \sqrt{\smash [b]{(\Delta \eta )^2 + (\Delta \phi )^2}}$, where $\Delta \phi$ ($\Delta \eta$) is the difference in azimuthal angle (pseudorapidity) between the two boson candidates. These requirements reject jet pairings that are inconsistent with a Z or H decay. This selection is based on that of the previous ATLAS measurement in Ref. [11], loosened to increase the signal acceptance.

Events in which all pairings fail the criteria in Eq. (2) are retained to increase the size of the data set used to train various classifiers in the analysis. If a pairing in these events satisfies one of the aforementioned requirements, it is chosen. When multiple pairings pass the same number of requirements, one is chosen randomly to give each event a location in the $m_{\textrm{jj}}^{\text {lead}}$-$m_{\textrm{jj}}^{\text {subl}}$ plane without biasing the background distribution.

**Fig. 1:**
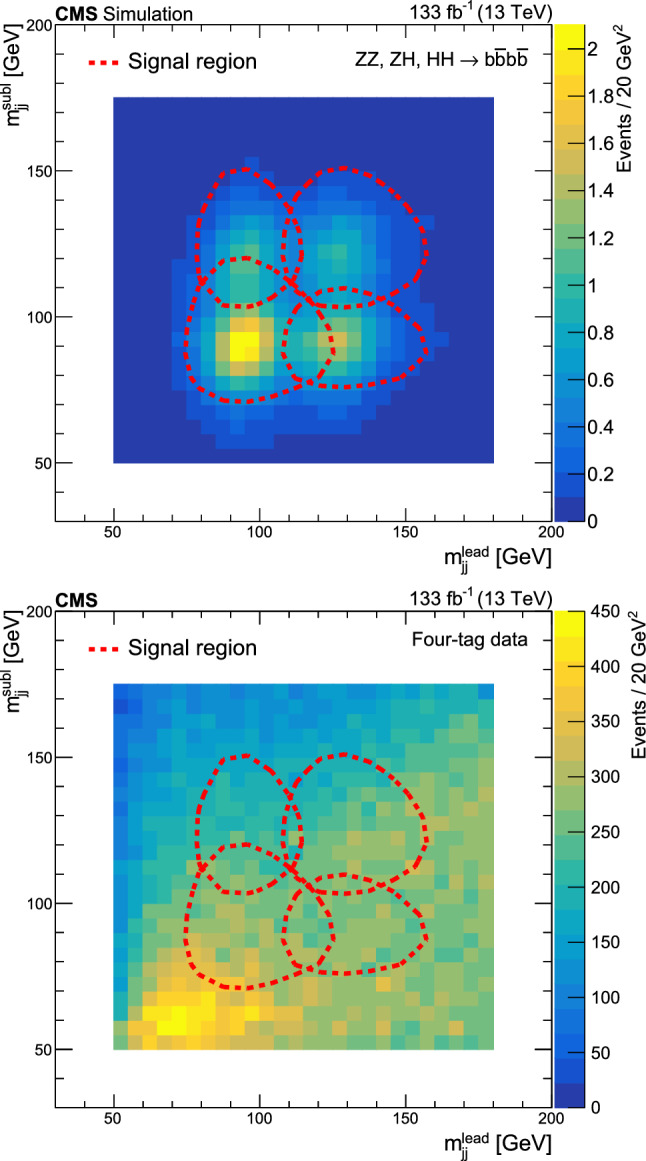
Signal yield from simulation (upper) and from four-tag events in data (lower), as a function of $m_{\textrm{jj}}^{\text {lead}}$and $m_{\textrm{jj}}^{\text {subl}}$. The color scale to the right of each plot gives the range of values. The signal region is defined by the union of the regions enclosed by the dashed red contours

The SR is defined using four overlapping regions in the dijet mass plane targeting ${\text {Z}{}{}} {\text {Z}{}{}}$, ${\text {Z}{}{}} {\text {H}{}{}}$, and ${\text {H}{}{}} {\text {H}{}{}}$ decays. These regions are defined using variables $X_{{{\text {Z}{}{}} {\text {Z}{}{}}}}$, $X_{{{\text {Z}{}{}} {\text {H}{}{}}}}$, $X_{{{\text {H}{}{}} {\text {Z}{}{}}}}$, and $X_{{{\text {H}{}{}} {\text {H}{}{}}}}$, defined as

3 $$\begin{aligned} X_{\textrm{B}_1 \textrm{B}_2} = \sqrt{ \Bigg (\frac{m_{\textrm{jj}}^{\text {lead}}- m_{\mathrm {B_1}}}{\sigma _{m_{\textrm{jj}}^{\text {lead}}}}\Bigg )^2 + \Bigg (\frac{m_{\textrm{jj}}^{\text {subl}}- m_{\mathrm {B_2}}}{\sigma _{m_{\textrm{jj}}^{\text {subl}}}}\Bigg )^2}, \end{aligned}$$

where $m_{\mathrm {B_1}}$ and $m_{\mathrm {B_2}}$ correspond to the nominal Z or H masses, corrected for the bias mentioned above, depending on the signal targeted. The denominators in each term is the approximate mass resolution, estimated from simulation to be 10% of the reconstructed boson mass. The SR is defined as the union of the requirements: $X_{{{\text {Z}{}{}} {\text {Z}{}{}}}} <2.6$, $X_{{{\text {Z}{}{}} {\text {H}{}{}}}} <1.9$, $X_{{{\text {H}{}{}} {\text {Z}{}{}}}} <1.9$, and $X_{{{\text {H}{}{}} {\text {H}{}{}}}} <1.9$. Figure 1 shows signal yield from simulation normalized to the expected yield (upper) and the four-tag events from data (lower), as a function of $m_{\textrm{jj}}^{\text {lead}}$ and $m_{\textrm{jj}}^{\text {subl}}$. The four regions used to define the SRs are shown by the red dashed contours.

**Fig. 2:**
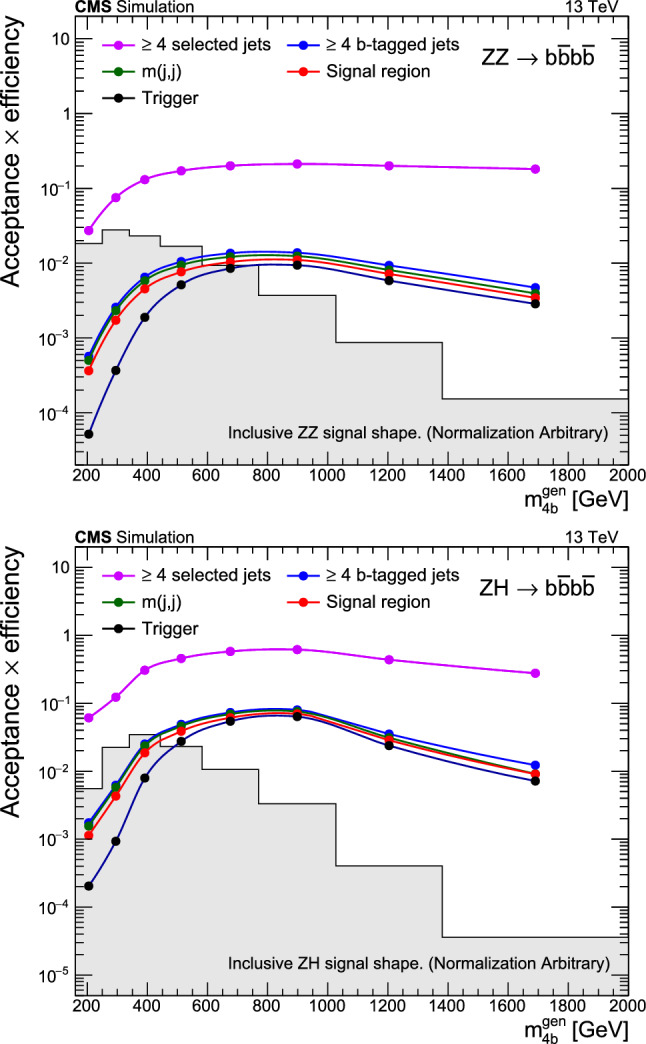
Event selection acceptance times efficiency as a function of the generated four-body mass $m_{4{\text {b}{}{}}}^{\text {gen}}$ for the ${\text {Z}{}{}} {\text {Z}{}{}}$ (upper) and ${\text {Z}{}{}} {\text {H}{}{}}$ (lower) signals. The plots show the cumulative efficiency with respect to the inclusive sample. The expected $m_{4{\text {b}{}{}}}^{\text {gen}}$ distributions of the inclusive ${\text {Z}{}{}} {\text {Z}{}{}}$ and ${\text {Z}{}{}} {\text {H}{}{}}$ events are shown by the gray-shaded areas with arbitrary normalization

The fractions of ${\text {Z}{}{}} {\text {Z}{}{}}$ and ${\text {Z}{}{}} {\text {H}{}{}}$ signal events within the detector acceptance from simulation, multiplied by the efficiency of each selection step are shown in the upper and lower plots of Fig. 2, respectively, as a function of the true four-body invariant mass, $m_{4{\text {b}{}{}}}^\text {gen}$. The cumulative effect of each selection requirement is shown for the ${\text {Z}{}{}} {\text {Z}{}{}}$ and ${\text {Z}{}{}} {\text {H}{}{}}$ channels. The product of the acceptance and efficiency is limited at low mass by the $H_{\textrm{T}}$ and jet $p_{\textrm{T}}$ requirements in the trigger. At high mass, the acceptance for resolving four jets drops and b tagging efficiency decreases for high $p_{\textrm{T}}$ jets. The trigger requirement significantly limits the efficiency at lower masses. The larger cross section of the ${\text {Z}{}{}} {\text {Z}{}{}}$ process compensates for the lower acceptance, leading to similar expected event yields.

The output from a multivariate classifier, described in Sect. 5, is used as the final discriminant between the signal and background. The classifier, referred to as the signal-versus-background (SvB) classifier, is trained to output the probability that an event is from one of five classes: multijet, ${{\textrm{t}}{}{}} {}{\bar{\textrm{t}}{}{}}$, ${\text {Z}{}{}} {\text {Z}{}{}}$, ${\text {Z}{}{}} {\text {H}{}{}}$, and ${\text {H}{}{}} {\text {H}{}{}}$. The probability of a signal event, defined as the sum of the probabilities of the event being from the ${\text {Z}{}{}} {\text {Z}{}{}}$, ${\text {Z}{}{}} {\text {H}{}{}}$, or ${\text {H}{}{}} {\text {H}{}{}}$ sample, is used in the final signal extraction.

Orthogonal ${\text {Z}{}{}} {\text {Z}{}{}}$ and ${\text {Z}{}{}} {\text {H}{}{}}$ SRs are defined according to which of the signal probabilities is the largest. A combined fit is performed in the ${\text {Z}{}{}} {\text {Z}{}{}}$ and ${\text {Z}{}{}} {\text {H}{}{}}$ regions to events with a signal probability larger than 1%, to extract the ${\text {Z}{}{}} {\text {Z}{}{}}$ and ${\text {Z}{}{}} {\text {H}{}{}}$ fitted signal strengths. The ${\text {H}{}{}} {\text {H}{}{}}$ results are not reported here as a dedicated measurement has been published in Ref. [4].

## Hierarchical combinatorial residual network

A multivariate classifier is used both for signal-versus-background discrimination as well as for the derivation and validation of the background model. An architecture, referred to as the hierarchical combinatorial residual (HCR) network, is specifically developed for the four-jet diboson topology. The HCR consists of a series of convolutional neural networks, each employing phase-symmetric convolutional filters [74] and residual learning [75], to process kinematic information from the boson-candidate jets; information from additional jets in the event is included using an attention block [76]. This network architecture provides a clean solution to the combinatorial jet-pairing problem, allowing the same set of weights to be optimized for all pairings. The convolutional layers are arranged hierarchically, first processing a jet image to form a dijet image, then processing the dijet image to form a quadjet image.

**Fig. 3:**
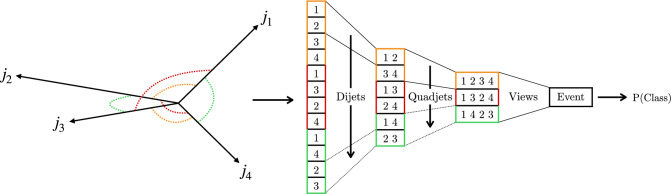
A high-level sketch of the HCR classifier architecture. Boson-candidate jets are shown on the left with the three possible jet pairings. The HCR architecture is shown on the right. The boxes represent pixels, with the labels indicating which jet, dijet, or quadjet the pixel refers to. The different jet pairings on the left are each represented within the network, as indicated by the color coding. The output P(class) corresponds to the the probability that an event belongs to the corresponding class used in training

Figure 3 shows a high-level sketch of the HCR architecture. The initial jet image is formed from pixels representing each of the boson-candidate jets, using the jet $p_{\textrm{T}},\,\eta ,\,\phi ,$ and mass values as features. Three copies of the jet pixels are arranged to form a one-dimensional image such that pairs of adjacent pixels represent the three possible jet pairings. Adjacent pairs of jet pixels are convolved to form a dijet image using a single set of weights. The second layer processes the six dijet pixels to form a three-pixel quadjet image. These three pixels are then combined to produce a single event-level pixel. Each convolution projects the input image into an eight-dimensional space; the dimension of this embedded space is a hyperparameter that controls the size of the network. A final output layer projects these features into a $N_{c}$-dimensional space, where $N_{c}$ is the number of input classes used in training. These outputs are converted into the probabilities that a given event belongs to the corresponding class used in training by applying softmax function.

Classifiers with the HCR architecture are used throughout the analysis. The SvB classifier employs the HCR architecture to construct the variable used in the final signal extraction. The HCR classifiers are also used to define the background model (Sect. 6) and in the construction of the synthetic data sets (Sect. 7.1).

## Background model

From simulation, it is found that 95% of the expected background consists of multijet events, which are modeled using data. The remaining 5% are ${{\textrm{t}}{}{}} {}{\bar{\textrm{t}}{}{}}$ events, which are modeled with simulation. Backgrounds from other sources, including processes involving single- and double-H production, are found to have a negligible contribution.

The QCD multijet background is modeled with an independent data set selected using the same trigger and selection requirements as used in the SR, except for the b tagging requirement: at least four jets with $p_{\textrm{T}} > 40\,\text {Ge}\hspace{-.08em}\text {V}$ are required, exactly three of which are required to be b tagged. To increase the size of this three-tag control sample, the b tagging requirement is loosened such that the efficiency to correctly identify a b jet is 70–80% depending on the data set. The size of three-tag sample is about 17 times larger than that of the four-tag sample. The three-tag sample consists of 90% multijet events and 10% ${{\textrm{t}}{}{}} {}{\bar{\textrm{t}}{}{}}$ events. Simulations indicate that the three-tag sample has a negligible contribution of signal events.

A product of two weights is applied to the three-tag events to model the multijet background in the four-tag sample. The first weight, referred to as the jet combinatorial model (JCM) weight, accounts for additional jet activity in the four-tag sample. The second weight, referred to as the four-vs-three (FvT) weight, corrects for kinematic differences between the three- and four-tag samples.

The weights are derived using a signal-depleted sideband (SB) region of the $m_{\textrm{jj}}^{\text {lead}}-m_{\textrm{jj}}^{\text {subl}}$ plane, shown in Fig. 1. The SB is defined as the region inside the mass window defined by Eq. (1) but outside the SR. The boundaries of the SB region are chosen to provide sufficient statistical precision, while ensuring that the kinematic properties of events in the SB are representative of those in the SR.

### Jet combinatorial model

The four-tag sample has a larger jet multiplicity than the three-tag sample, since the requirement of exactly three b-tagged jets in the three-tag sample biases the jet multiplicity. This effect is modeled using an extension of the combinatorial technique introduced in Ref. [11]. For each three-tag event, all possible combinations of jets not satisfying the looser b tagging requirement, referred to as anti-b-tagged jets, are considered. At least one anti-b-tagged jet is treated as a b jet. The anti-b-tagged jets that are treated as b jets are referred to as pseudo-tagged jets. A constant per-jet transfer factor *f* is assigned to each pseudo-tagged jet and a factor $(1-f)$ to the remaining anti-b-tagged jets. Correlations in the transfer factor among jets arise because b jets are produced in pairs. These correlations are modeled with a pair-enhancement term *e*, that is included in combinations with an even number of tagged plus pseudo-tagged jets. This enhancement term is expected to be suppressed at higher jet multiplicities since the probability of observing an odd number of b-tagged jets increases because of mistagging and jets falling outside of the detector acceptance. The pair-enhancement factor is thus divided by $n^d$, where *n* is the number of anti-b-tagged jets and *d* is a free parameter that controls the suppression of *e* with jet multiplicity. Finally, an overall normalization *t* is included to account for the looser b tagging requirement in the three-tag sample. The per-event JCM weight is thus computed as:

4 $$\begin{aligned}{} & {} w_{\text {JCM}} \nonumber \\{} & {} \quad = {\left\{ \begin{array}{ll} t\sum _{i=1}^{n} \left( {\begin{array}{c}n\\ i\end{array}}\right) f^{i}(1-f)^{n-i} (1+ \textrm{e}/n^d) &{} (3+i)\quad \text {even} \\ t\sum _{i=1}^{n} \left( {\begin{array}{c}n\\ i\end{array}}\right) f^{i}(1-f)^{n-i}&(3+i)\quad \text {odd}, \end{array}\right. } \nonumber \\ \end{aligned}$$

where *i* is the number of pseudo-tagged jets and $$\left( {\begin{array}{c}n\\ i\end{array}}\right) $$ is the binomial coefficient.

**Fig. 4:**
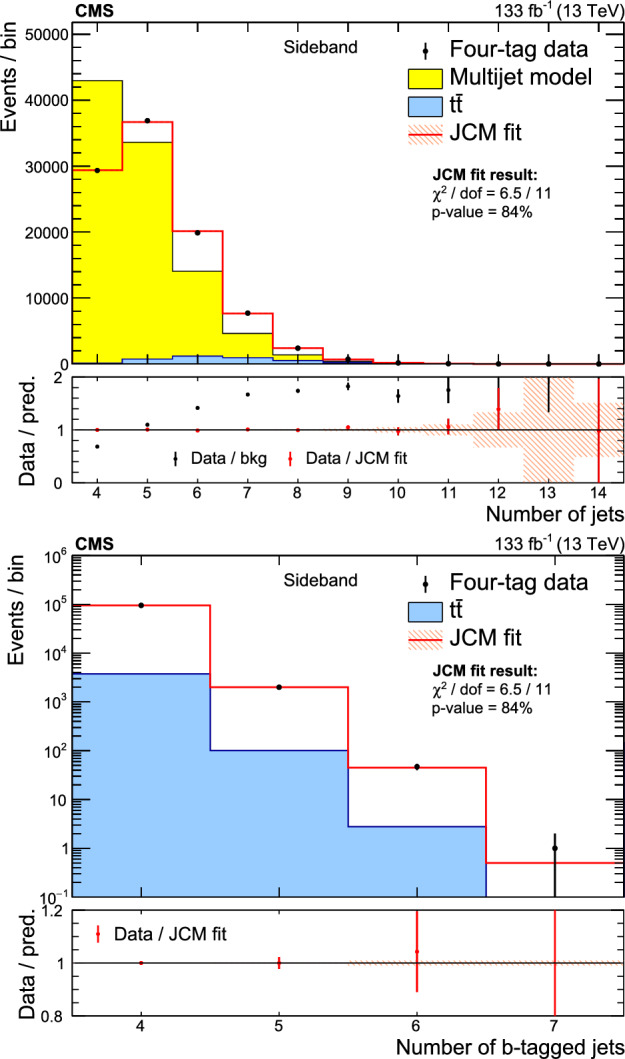
Jet (upper) and b-tagged jet (lower) multiplicity distributions in the SB region. The black data points show the observed four-tag data, the blue distribution the ${{\textrm{t}}{}{}} {}{\bar{\textrm{t}}{}{}}$ simulation, and the yellow histogram the three-tag multijet prior to the JCM corrections. The red histogram shows the result of the fit to the JCM model. The quality of the fit is given by the $\chi ^2$ per degrees of freedom (dof) and corresponding *p*-value in the legend. The lower panels display the ratio of the data to the fit prediction

The JCM parameters are determined by a combined fit to the jet and b-tagged jet multiplicity distributions in the SB region. Selected jets are required to satisfy the same kinematic requirements, $p_{\textrm{T}} > 40$ $\,\text {Ge}\hspace{-.08em}\text {V}$ and $|\eta | < 2.4$, as the boson-candidate jets. The jet and b-tagged jet multiplicity distributions are shown in Fig. 4 (upper) and (lower), respectively, along with the results of the fit. The black data points show the observed four-tag data; the yellow distribution displays the multijet background estimate prior to the JCM correction, and the blue distribution is from the ${{\textrm{t}}{}{}} {}{\bar{\textrm{t}}{}{}}$ simulation. The multijet background prior to the JCM correction is given by the ${{\textrm{t}}{}{}} {}{\bar{\textrm{t}}{}{}}$-subtracted three-tag data, normalized to the ${{\textrm{t}}{}{}} {}{\bar{\textrm{t}}{}{}}$-subtracted four-tag data. This component is not shown on the lower panel as the three-tag data cannot be used to model the b-tagged jet multiplicity. The red histogram shows the result of the JCM fit, which provides a good description of both the jet and b-tagged jet multiplicities.

### Kinematic reweighting

**Fig. 5:**
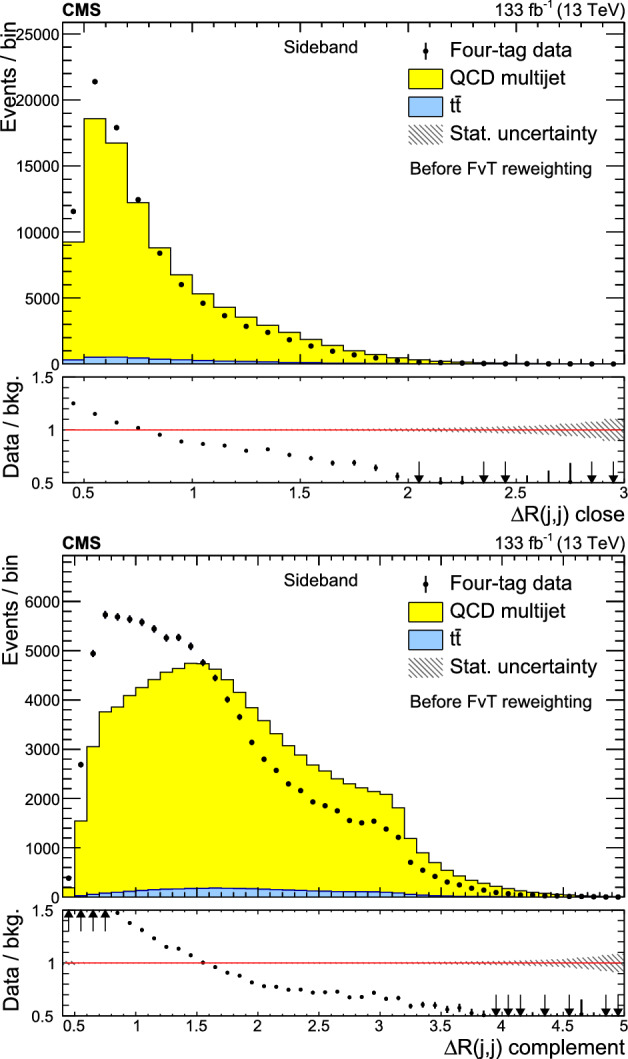
Distributions of $\Delta R(j, j)_{\text {close}}$ (upper) and $\Delta R(j, j)_{\text {complement}}$ (lower). The four-tag SB events are shown by the points. The QCD multijet distribution (yellow region) is from the three-tag SB sample after the JCM correction but before the FvT kinematic reweighting, and the ${{\textrm{t}}{}{}} {}{\bar{\textrm{t}}{}{}}$ distribution (blue region) is from simulation. The lower panels display the ratio of the four-tag data to the total background, which is the sum of the QCD multijet and ${{\textrm{t}}{}{}} {}{\bar{\textrm{t}}{}{}}$ distributions. The hatched area gives the statistical uncertainty in the background

**Fig. 6:**
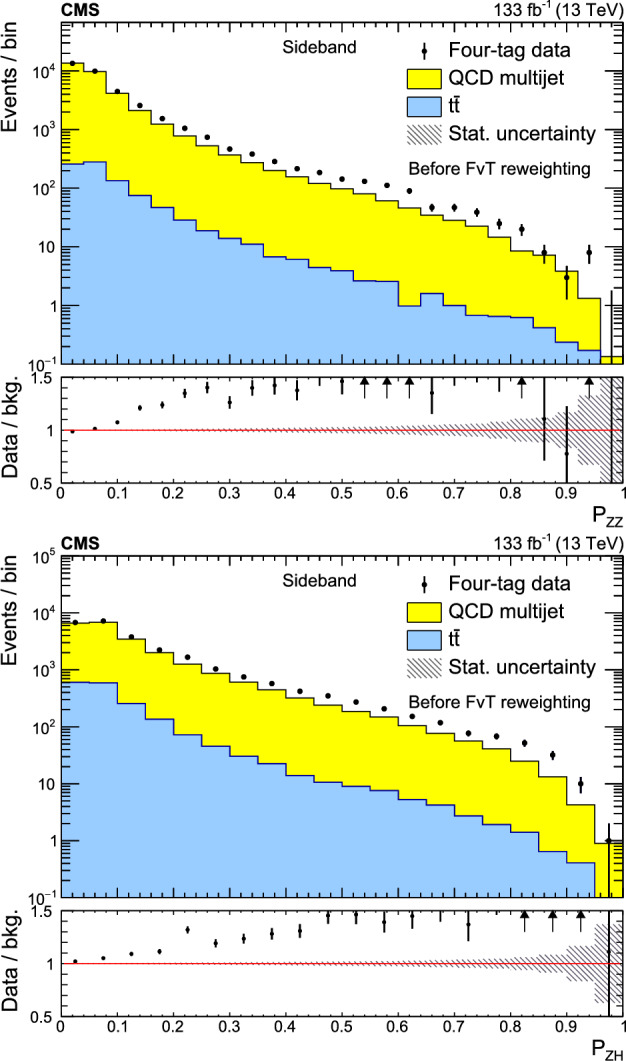
Distributions of the signal probabilities for ${\text {Z}{}{}} {\text {Z}{}{}}$ (upper) and ${\text {Z}{}{}} {\text {H}{}{}}$ (lower) in the SB region, respectively. The four-tag SB events are shown by the points. The QCD multijet distribution (yellow region) is from the three-tag SB sample after the JCM correction but before the FvT kinematic reweighting, and the ${{\textrm{t}}{}{}} {}{\bar{\textrm{t}}{}{}}$ distribution (blue region) is from simulation. The lower panels display the ratio of the four-tag data to the total background, which is the sum of the QCD multijet and ${{\textrm{t}}{}{}} {}{\bar{\textrm{t}}{}{}}$ distributions. The hatched area gives the statistical uncertainty in the background

After correction with the JCM weights, differences remain between the three- and four-tag samples arising from the kinematic dependence of the b tagging efficiency and from a different mixture of underlying scattering processes in the two selections. These differences are highlighted in Fig. 5, which shows the opening angles $\Delta R(j, j)$ for dijet pairs, chosen such that the boson-candidate jets with the smallest opening angle form one pair (close dijet) and the remaining boson-candidate jets form the other (complement dijet). The opening angles are shown for events in the SB region. The multijet model, shown in yellow, includes the JCM weights. The four-tag sample is dominated by gluon splitting to ${\text {b}{}{}} {\bar{{\text {b}{}{}}}{}{}}$ from an underlying two-to-two scattering process. This produces a topology with two b-tagged dijets, each with a small $\Delta \text {R}(j,j)$. The three-tag sample contains this process in addition to a mixture of processes where the anti-b-tagged boson-candidate jet can be produced without gluon splitting, leading to a broader distribution of opening angles. Significant differences between the three- and four-tag samples are also observed in other jet, dijet, and event-level distributions. Figure 6 shows the modeling of the final discriminating variables – the SvB signal probabilities for ${\text {Z}{}{}} {\text {Z}{}{}}$ ($\textrm{P}_{{\text {Z}{}{}} {\text {Z}{}{}} }$) and ${\text {Z}{}{}} {\text {H}{}{}}$ ($\textrm{P}_{{\text {Z}{}{}} {\text {H}{}{}} }$) – in the SB region.

The residual kinematic differences between the three- and four-tag samples are corrected using weights derived from a multivariate classifier with the HCR architecture. This classifier, referred to as the FvT classifier, is trained with four classification targets: four-tag data, four-tag ${{\textrm{t}}{}{}} {}{\bar{\textrm{t}}{}{}}$ simulation, three-tag data, and three-tag ${{\textrm{t}}{}{}} {}{\bar{\textrm{t}}{}{}}$ simulation. The FvT classifier is trained using data and the ${{\textrm{t}}{}{}} {}{\bar{\textrm{t}}{}{}}$ simulation in the SB region. The JCM weights are applied to the three-tag data and three-tag ${{\textrm{t}}{}{}} {}{\bar{\textrm{t}}{}{}}$ simulation prior to training. The output probabilities are used to reweight the three-tag data to the four-tag multijet background. The FvT weights are given by

5 $$\begin{aligned} w_{\text {FvT}} = \frac{P(\text {M}_{\textrm{4b}})}{P(\text {D}_{\textrm{3b}})} \equiv \frac{P(\text {D}_{\textrm{4b}})- P({{{\textrm{t}}{}{}} {}{\bar{\textrm{t}}{}{}} }_{\textrm{4b}})}{P(\text {D}_{\textrm{3b}})}, \end{aligned}$$

where $P(\text {D}_{\textrm{4b}})$, $P({{{\textrm{t}}{}{}} {}{\bar{\textrm{t}}{}{}} }_{\textrm{4b}})$, and $P(\text {D}_{\textrm{3b}})$ are the class-assignment probabilities coming from the FvT classifier output, and $\text {M}_{\text {4b}}$ refers to the QCD multijet four-tag event. The final background model is given by the four-tag ${{\textrm{t}}{}{}} {}{\bar{\textrm{t}}{}{}}$ simulation plus the three-tag data weighted by the product of the JCM and FvT weights.

**Fig. 7:**
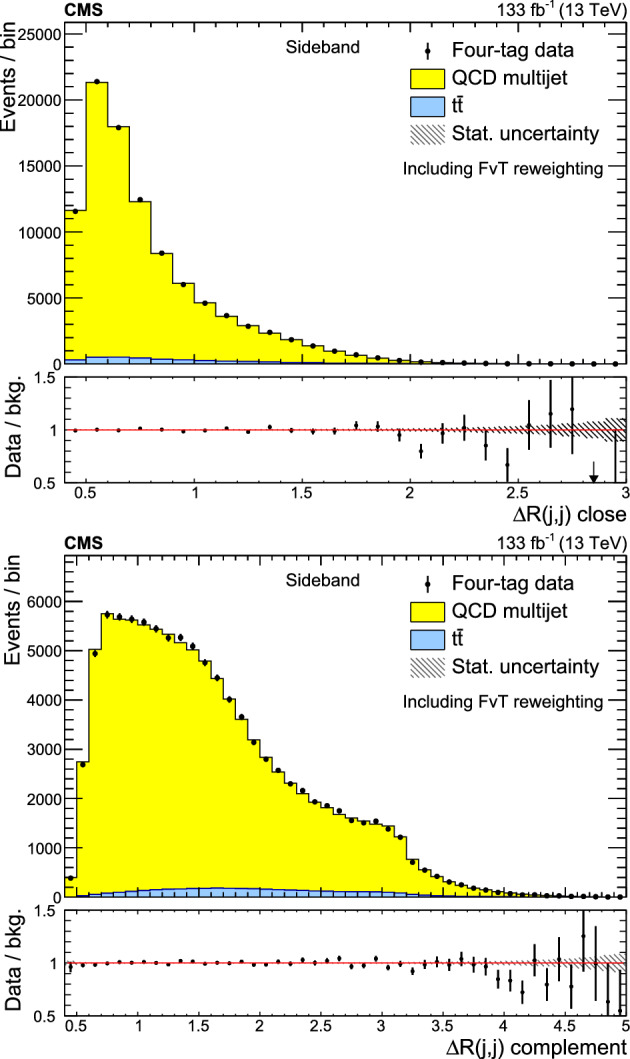
The $\Delta \text {R}(j,j)$ distributions shown in Figure 5 after including the FvT corrections to the QCD multijet prediction

**Fig. 8:**
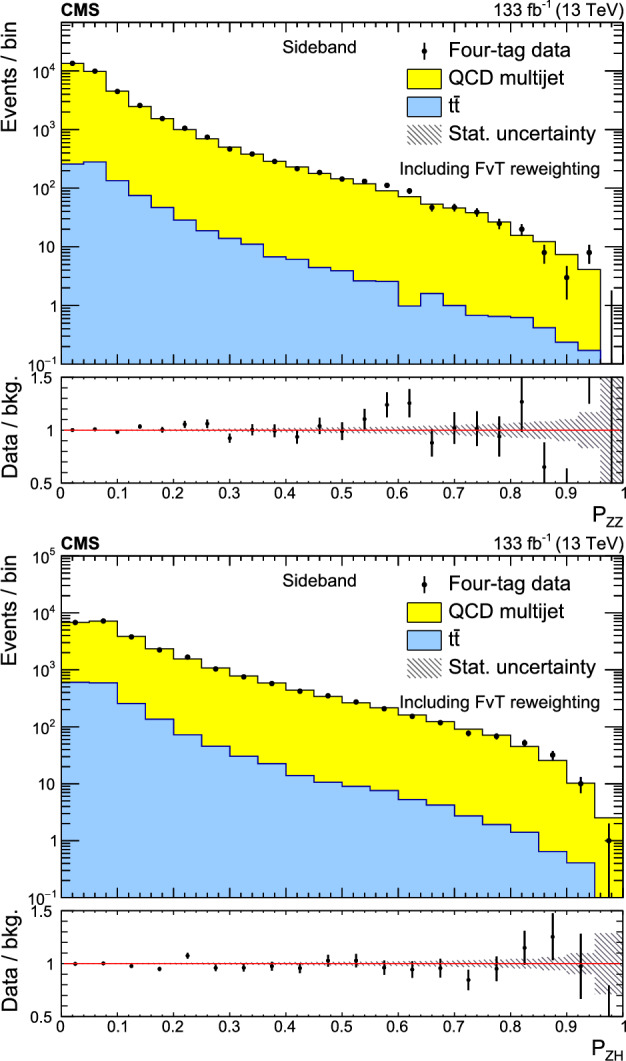
Distribution of signal probabilities for ${\text {Z}{}{}} {\text {Z}{}{}}$ (upper) and ${\text {Z}{}{}} {\text {H}{}{}}$ (lower) events in the SB region after including the FvT corrections to the QCD multijet prediction

Figure 7 shows the improvement in modeling of the close and complement candidate opening angles when the FvT weights are applied. The FvT weights also correct the modeling of the other observed discrepancies in jet, dijet, and event-level distributions. The impact of the FvT reweighting on the modeling of the SvB signal probabilities in the SB region is shown in Fig. 8.

## Background validation

The accurate modeling of the SvB signal probabilities in Fig. 8 comes with two major caveats. The first is that the FvT classifier is trained with the four-tag SB region data and thus has access to all of the relevant SvB information during training. The final analysis in the SR requires an extrapolation of the background model to a different region of phase space. This extrapolation is a significant source of systematic uncertainty that cannot be assessed in the region used to train the classifier. The second major caveat is that the sensitivity in the SR is dominated by SvB signal probabilities above ${\approx }$ 0.9, for which there is little statistical power in the SB region.

These problems are addressed by validating the background model with synthetic data sets that allow the extrapolation of the background model to be tested precisely in regions of high signal probability. Section 7.1 describes the construction of the synthetic data sets, and Sect. 7.2 describes how it is used to assess the systematic uncertainties in the background model.

### Synthetic data sets from hemisphere mixing

A synthetic data set is generated by splitting individual events into hemispheres and then combining similar hemispheres from different events. This mixing procedure suppresses correlations among the jet four-vectors due to the presence of a signal, while preserving the kinematic distributions of the 4b background. The relevant event-level correlations in the background – which primarily arise from gluon splitting in an underlying two-to-two scattering process – are captured in the correlation between hemispheres and are independent of the dijet substructure within the hemispheres.

The mixing algorithm is based on the technique developed in a previous CMS ${\text {H}{}{}} {\text {H}{}{}} \rightarrow 4{\text {b}{}{}}$ analysis [20]. The first step involves creating a collection of hemispheres (hemisphere library) from events in the four-tag data set. Each event is split into two hemispheres using the plane orthogonal to the transverse thrust axis [20], which is chosen based on the assumption that it is a good proxy for the initial gluon directions in the underlying scattering process. Jets on one side of the plane are assigned to one hemisphere, those on the other side are assigned to the other hemisphere. Four variables are computed using the sum of the four-vectors of all the jets in that hemisphere: the invariant mass, the longitudinal momentum, and the transverse momentum perpendicular and parallel to the transverse thrust axis. The jet and b jet multiplicities are also computed for each hemisphere. The library is created with events that pass the jet kinematic requirements but before the dijet invariant mass requirement given in Eq. (1) is applied.

The mixing is performed in a second pass over the data. Events are split into hemispheres as before, and the corresponding hemisphere summary variables are calculated. Each hemisphere in the event is replaced with its nearest neighbor in the hemisphere library. Nearest neighbors are defined as hemispheres with the same jet and b jet multiplicities that minimize the distance between hemispheres, defined as

6 $$\begin{aligned} d(h_i,h_j) = \sqrt{\sum _{k} \left( \frac{v_{k}(h_i) - v_{k}(h_j)}{\sigma _{v_{k}}}\right) ^2}, \end{aligned}$$

where $h_i$ and $h_j$ are two hemispheres and the sum runs over the four hemisphere summary variables $v_k$ and $\sigma _{v_{k}}$ is the root mean square of the corresponding variable calculated from all hemispheres in the library. During the nearest neighbor replacement a check is made that the matching hemispheres do not come from the same event. Finally, the nearest neighbor hemispheres are rotated in the azimuthal angle $\phi$ to match the direction of the transverse thrust axis of the input event.

**Fig. 9:**
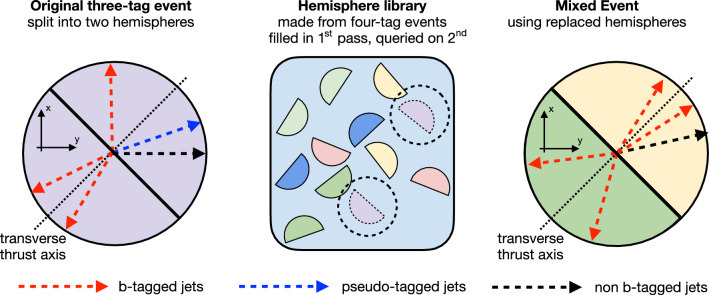
An illustration of the hemisphere mixing procedure, adapted from Ref. [20]. Three-tag events are divided into two halves by cutting along the axis perpendicular to the transverse thrust axis. In a preliminary step, each event in the four-tag data set is split into two hemispheres that are collected in a library of hemispheres. Once the library is created, each three-tag event is used as a basis for creating a synthetic event. These are constructed by picking the two hemispheres from the library that are most similar to the hemispheres making up the original event

This analysis introduces two important improvements to the mixing strategy. The first is the use of the three-tag data set in mixing. Four-tag events are used to create the hemisphere library, however, the three-tag data set is used in the second pass when creating the mixed data sample. In the three-tag sample, the pseudo-tagged jets are treated as b tagged when matching hemispheres; this ensures that events in the resulting mixed data sample all have four b-tagged jets. An illustration of the mixing procedure is given in Fig. 9. Mixing the three-tag data eliminates a potential bias from signal contamination and allows for the construction of a synthetic data sample that is fifteen times larger than the four-tag data sample.

The other important improvement to the mixing algorithm is the introduction of corrections accounting for the ${{\textrm{t}}{}{}} {}{\bar{\textrm{t}}{}{}}$ background contamination. The presence of the ${{\textrm{t}}{}{}} {}{\bar{\textrm{t}}{}{}}$ events complicates the mixing strategy, since these events have significant hemisphere-level correlations coming from the decays of the top quarks. Mixing a ${{\textrm{t}}{}{}} {}{\bar{\textrm{t}}{}{}}$ hemisphere with a QCD multijet hemisphere would significantly distort the ${{\textrm{t}}{}{}} {}{\bar{\textrm{t}}{}{}}$ background. The mixed ${{\textrm{t}}{}{}} {}{\bar{\textrm{t}}{}{}}$ events are thus not expected to provide a good model of the unmixed ${{\textrm{t}}{}{}} {}{\bar{\textrm{t}}{}{}}$ background.

The ${{\textrm{t}}{}{}} {}{\bar{\textrm{t}}{}{}}$ background needs to be subtracted from the hemisphere library and the three-tag data set being mixed – not from projections into histograms, as is more typical in high energy physics. These ${{\textrm{t}}{}{}} {}{\bar{\textrm{t}}{}{}}$ contributions are subtracted statistically using event weights derived from a multivariate classifier with the HCR architecture. This classifier is trained with two classes, data and ${{\textrm{t}}{}{}} {}{\bar{\textrm{t}}{}{}}$ simulation, separately on events in the three- and four-tag data set. The outputs from the classifier are used to determine the probability $P(\text {M})$ that an event in the three- or four-tag data sample is a multijet event. When constructing the mixed data sample, an input three-tag event is accepted for mixing with probability $P(\text {M})$. Similarly, nearest-neighbor hemispheres are accepted as replacements with probability $P(\text {M})$, as calculated in their corresponding four-tag event. When a hemisphere is rejected, the next-nearest neighbor is considered in turn.

The size of the four-tag data sample in the SB region, used to train the FvT classifier, is a fundamental limitation of the background determination procedure. When validating the background, it is critical that the synthetic data have the same statistical power as the nominal four-tag sample. To ensure this, the mixed data are subsampled to match the size of the QCD multijet background in the four-tag sample. The relative size of the mixed data sample allows fifteen independent subsamples to be created. The number of subsamples is somewhat smaller than the relative size of the three-tag dataset to avoid using duplicate events with large jet multiplicity. Events from the four-tag ${{\textrm{t}}{}{}} {}{\bar{\textrm{t}}{}{}}$ simulation are added to each subsample according to the expected ${{\textrm{t}}{}{}} {}{\bar{\textrm{t}}{}{}}$ yield. These fifteen synthetic data sets are referred to as mixed models.

**Fig. 10:**
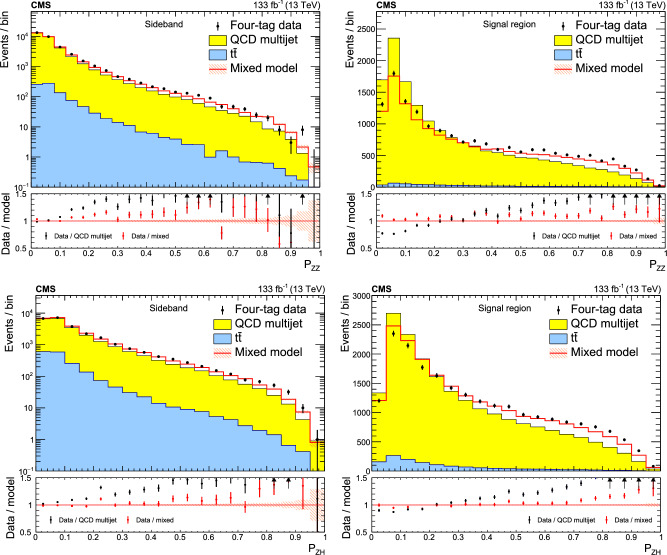
Distribution of signal probabilities for ${\text {Z}{}{}} {\text {Z}{}{}}$ (upper row) and ${\text {Z}{}{}} {\text {H}{}{}}$ (lower row) events in the sideband (left) and signal regions (right). The four-tag events are shown by the points. The QCD multijet distribution before the FvT corrections is given by the yellow region, and the simulated ${{\textrm{t}}{}{}} {}{\bar{\textrm{t}}{}{}}$ distribution by the blue area. The average of the mixed models (red) provides a high-event-count proxy of the 4b background (black) that allows the extrapolation of the background model to be tested precisely. The lower panels display the ratio of the four-tag data to the average of the mixed models (red) and to the QCD multijet distribution (black)

The advantages of using the mixed models to validate the background can be seen in Fig. 10. The ${\text {Z}{}{}} {\text {Z}{}{}}$ (${\text {Z}{}{}} {\text {H}{}{}}$) SvB signal probabilities are shown in the upper (lower) figures in the SB region, left, and in the SR, right. The four-tag data are shown by the black points. The three-tag data distribution before applying the kinematic FvT corrections, from which the background prediction is extrapolated, is shown in yellow. The average of the mixed models, shown in red, provides a high-event-count proxy for the four-tag background that can be used to validate the background prediction in regions of high signal probability.

### Background model uncertainties

The background procedure, as described in Sect. 6, is repeated by treating the mixed models as four-tag data. The procedure is performed separately using each of the fifteen mixed models. Differences among the fifteen QCD multijet predictions and the comparison of the predicted background to the observed SR yield are used to assign systematic uncertainties in the nominal background model. These systematic uncertainties are assessed in three steps. First, the differences among the fifteen QCD multijet predictions are quantified. These differences arise from the finite size of the data sample in the SB region used to train the FvT classifier. The second step measures the systematic uncertainty from extrapolating to the SR, by comparing the background predictions to the observed yields in the mixed models. The final step checks if biases in the background model can mimic a signal. This process is carried out independently for the final ${\text {Z}{}{}} {\text {Z}{}{}}$ and ${\text {Z}{}{}} {\text {H}{}{}}$ selections.

The differences in the background models are quantified by comparing the QCD multijet predictions of each mixed model in the SR to their average. These differences are parameterized by a set of orthogonal Fourier basis functions added to the background predictions. Each of the multijet predictions, with unconstrained coefficients for the basis function corrections, are fit separately to the average. Basis functions with increasing frequency components are added until the pulls of adjacent SvB signal probability bins from all the mixed-model predictions are consistent with being uncorrelated. A pull is defined as the difference between the observed and expected values, divided by the uncertainty in the difference. A systematic uncertainty in each coefficient is assigned based on the root mean square of the fitted values. This uncertainty accounts for the expected variance of a single background prediction due to the finite size of the data sample in the SB region.

This procedure is carried out separately for the ${\text {Z}{}{}} {\text {Z}{}{}}$ and ${\text {Z}{}{}} {\text {H}{}{}}$ signal probabilities. Five (four) basis functions, with uncertainties in the basis-function coefficients of up to 3%, are needed to characterize the differences among the ${\text {Z}{}{}} {\text {Z}{}{}}$ (${\text {Z}{}{}} {\text {H}{}{}}$) background predictions.

The systematic uncertainty from extrapolating the background prediction is evaluated by comparing the SR predictions to the observed yields in the mixed models. A combined background model is fit to the average of the observed SR yields. Averaging the fifteen mixed models improves the precision with which the extrapolation uncertainty can be determined. The combined background model consists of the estimated ${{\textrm{t}}{}{}} {}{\bar{\textrm{t}}{}{}}$, the average of the QCD multijet predictions, and the basis-function corrections determined above. The coefficients of the basis functions are treated as nuisance parameters constrained using the systematic uncertainties assigned in the previous step.

The extrapolation uncertainty is quantified using the basis-function coefficients determined from fitting the mixed models. The fit is repeated, sequentially removing constraints on the nuisance parameters, until the fit has a *p*-value greater than 5% and an F-test [77] does not prefer more unconstrained basis function coefficients. Nonzero fitted coefficients represent a systematic difference between the predicted and observed background. Systematic uncertainties in the background extrapolation are assigned by adding the magnitude of the fitted coefficients in quadrature with their uncertainty. These extrapolation uncertainties are treated as uncorrelated from the variance uncertainties assigned in the previous step.

**Fig. 11:**
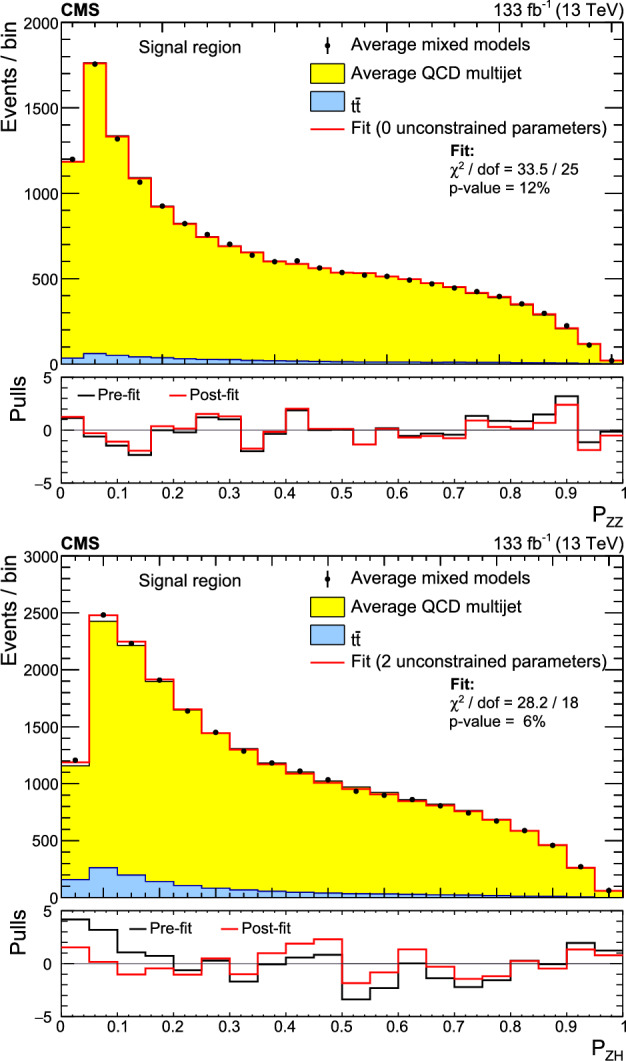
The distributions of the ${\text {Z}{}{}} {\text {Z}{}{}}$ (upper) and ${\text {Z}{}{}} {\text {H}{}{}}$ (lower) signal probabilities. The black data points show the average of the mixed models. The yellow and blue distributions show the average of the QCD multijet models and the ${{\textrm{t}}{}{}} {}{\bar{\textrm{t}}{}{}}$ simulation, respectively. The red histogram displays the post-fit results of the data fit to the background model. The ${\text {Z}{}{}} {\text {Z}{}{}}$ channel data distribution is fit with all five basic coefficients constrained, while the ${\text {Z}{}{}} {\text {H}{}{}}$ channel distribution has two of the four coefficients unconstrained. The lower panels give the pre- (blue) and post-fit (red) pulls

Figure 11 illustrates the process in determining the extrapolation uncertainty in the ${\text {Z}{}{}} {\text {Z}{}{}}$ (left) and ${\text {Z}{}{}} {\text {H}{}{}}$ (right) SRs. The upper panels compare the mixed model SvB signal probability in the SR, given by the black points, to the pre-fit background prediction, shown as stacked yellow and blue histograms, and the post-fit background prediction, shown in red. The lower panels show the pulls. For the ${\text {Z}{}{}} {\text {Z}{}{}}$ SR, none of the fit parameters need to be unconstrained to satisfy the goodness-of-fit or the F-test criteria. For the ${\text {Z}{}{}} {\text {H}{}{}}$ SR, the goodness-of-fit criteria is not satisfied until two parameters are unconstrained, at which point the F-test criterion is also satisfied. The extrapolation uncertainty is ${\lesssim }\ 1\%$ for most parameters and at most ${\approx }\ 3\% ({\approx }\ 5\%)$ in the ${\text {Z}{}{}} {\text {Z}{}{}}$ (${\text {Z}{}{}} {\text {H}{}{}}$) region.

The mixed models can also be used to test if biases in the background model can mimic the signature of a signal, a possibility to which many analyses with backgrounds estimated from control samples in data are blind. To assess the risk of fitting a spurious signal, the fit to the averaged mixed models in the second step is repeated with and without an unconstrained signal template. For this test, the coefficients of the basis functions in the background model are constrained with the systematic uncertainties assigned in the previous two steps. An F-test is performed that compares the background-only model to the model including an unconstrained signal template. In both SRs, it is found that allowing for a spurious signal does not lead to a significant improvement in the model fit, therefore no additional systematic uncertainty is assigned.

## Systematic uncertainties

A maximum likelihood fit to the four-tag data is performed on the distribution of the SvB signal probability simultaneously in the ${\text {Z}{}{}} {\text {Z}{}{}}$ and ${\text {Z}{}{}} {\text {H}{}{}}$ SRs. Systematic uncertainties are treated as nuisance parameters with either Gaussian (shape uncertainties) or log-normal (normalization uncertainties) function priors included in the likelihood function. All systematic uncertainties are considered as shape uncertainties with the exception of the luminosity, predicted signal cross section, and branching fraction uncertainties, which are treated as normalization uncertainties.

Table 1 summarizes the impact of different sources of uncertainty in the ${\text {Z}{}{}} {\text {Z}{}{}}$ and ${\text {Z}{}{}} {\text {H}{}{}}$ signal sensitivity. The table shows the relative contributions of the various sources of uncertainty in the measured signal strength, quoted in terms of a percentage of the total uncertainty. The contributions from the leading sources of uncertainty – background modeling, b tagging, and jet energy scale and resolution – are listed separately. The remaining uncertainties, described below, are included in the row labeled “Others”. The statistical uncertainty accounts for over half of the total uncertainty, while the remaining uncertainty is primarily due to experimental uncertainties in the background model and the b tagging efficiency.

**Table 1:** Summary of the relative uncertainties form the various sources in the measured signal strength, expressed as a percentage of the total uncertainty for the ${\text {Z}{}{}} {\text {Z}{}{}}$ and ${\text {Z}{}{}} {\text {H}{}{}}$ channels. The two uncertainties coming from the background modeling are given separately in parentheses, as well as their sum. The total systematic uncertainties shown include the effects of correlations

Source	${\text {Z}{}{}} {\text {Z}{}{}}$	${\text {Z}{}{}} {\text {H}{}{}}$
Statistical uncertainty	75	77
Total systematic uncertainty	67	64
Background model	61	56
(Variance)	(46)	(46)
(Extrapolation)	(40)	(33)
b tagging	9	17
Jet energy scale and resolution	9	5
Others	24	24

The uncertainties in the background model are described in Sect. 7. Similar results are obtained when characterizing the shape differences using a Fourier or a shifted Legendre polynomial basis. Despite being determined with a relative error of a few percent, the uncertainty in the background prediction accounts for ${\approx }60\%$ of the total uncertainty in the measured signal strengths.

The efficiency of the b tagging requirement in the simulated samples is corrected to match the efficiency measured in data [47]. These corrections, along with their corresponding uncertainties, are determined in bins of jet $p_{\textrm{T}}$, $\eta$, and the DeepJet b tagging score. The largest uncertainties in the b tagging efficiency arise from contamination of light-flavor jets in heavy-flavor control regions. To evaluate the impact of the b tagging uncertainties, the per-jet uncertainty in the b tagging corrections is propagated to the final SvB distribution. The b tagging uncertainties result in a roughly flat ${\pm }20\%$ variation in signal yield, with no significant variations in the SvB shape, and contribute 10–20% of the total uncertainty.

Uncertainties in the modeling of the jet energy scale and resolution, and the b jet energy scale correction in the simulation are estimated by propagating variations of the calibrations [41] to the final SvB discriminant distributions. These variations change the reconstructed energy and direction of simulated jets and can thus result in event migration across regions and signal probability bins. The combined jet energy and resolution uncertainties contribute 5–10% of the total uncertainty.

The efficiency of the trigger requirement in the simulated samples is adjusted to match the efficiency measured in data. The efficiencies for b jets to pass the various trigger thresholds, based on their $p_{\textrm{T}}$ and b tagging scores, are measured in a ${{\textrm{t}}{}{}} {}{\bar{\textrm{t}}{}{}}$ sample where both top quarks decay leptonically. Systematic uncertainties on the measured trigger efficiency are evaluated and applied to the expected signal yield. The largest trigger uncertainty comes from the calculation of per-event trigger efficiencies using the measured per-jet efficiencies. The total uncertainty in the trigger efficiency is estimated to be ${\approx }\ 5\%$ for both the ${\text {Z}{}{}} {\text {Z}{}{}}$ and ${\text {Z}{}{}} {\text {H}{}{}}$ signals.

The total uncertainty in the ${\text {Z}{}{}} {\text {Z}{}{}}$ (${\text {Z}{}{}} {\text {H}{}{}}$) cross section prediction is 6.6% (4.1%). These uncertainties include the effects of varying the renormalization and factorization scales and the parton distribution function (PDF) of the proton. The uncertainty from the choice of the factorization and renormalization scales in the calculation of the matrix element for the hard-scattering process is estimated by varying each scale by factors of 0.5 and 2, excluding anticorrelated combinations, to calculate the envelope around the central value. In order to estimate the impact on the results due to the uncertainty on the proton PDF, event weights corresponding to the different set of NNPDF [70] replicas are applied to the simulation. The uncertainty in the H $\rightarrow$ ${\text {b}{}{}} {\bar{{\text {b}{}{}}}{}{}}$ branching fraction is ±1.3% [22].

The uncertainty in the total integrated luminosity for each data set has been measured in Refs. [44–46]. A correlation scheme is used for the three sets of uncertainties based on correlated features in calibration methods, measurements, and data sets, resulting in an uncertainty of 1.6% for the full data set.

**Fig. 12:**
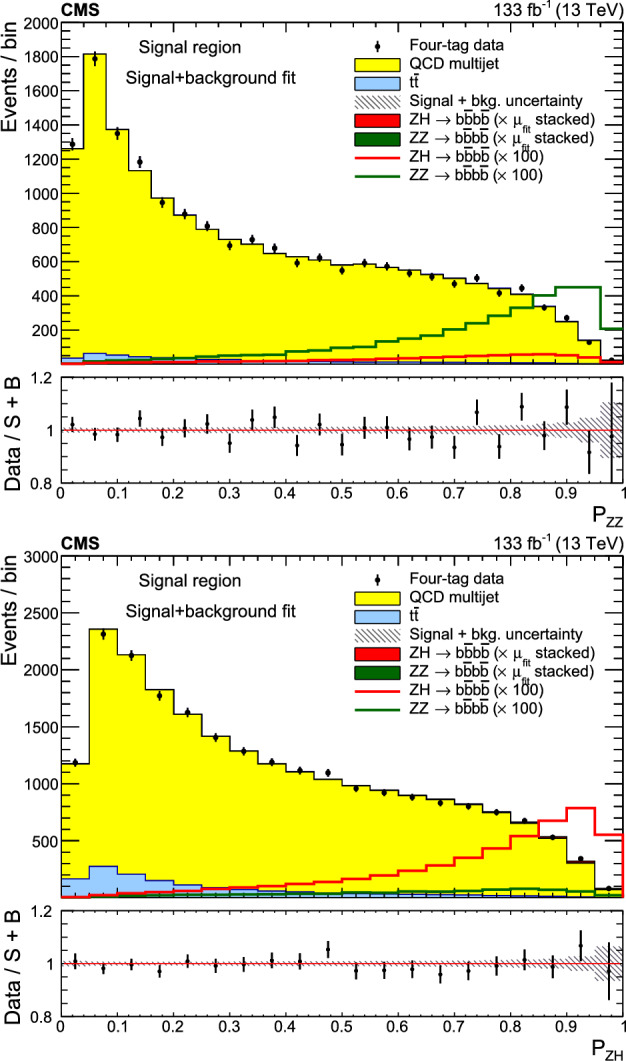
Distributions of signal probabilities for ${\text {Z}{}{}} {\text {Z}{}{}}$ (upper) and ${\text {Z}{}{}} {\text {H}{}{}}$ (lower) channels (points), along with the post-fit QCD multijet (yellow region) plus ${{\textrm{t}}{}{}} {}{\bar{\textrm{t}}{}{}}$ (blue region) distributions. The ${\text {Z}{}{}} {\text {H}{}{}}$ and ${\text {Z}{}{}} {\text {Z}{}{}}$ signal distributions scaled to the fitted signal strengths are shown, stacked on top of the background prediction. The expected ${\text {Z}{}{}} {\text {H}{}{}}$ (red histograms) and ${\text {Z}{}{}} {\text {Z}{}{}}$ (green histograms) signal channel distributions are also shown separately, multiplied by 100 for visibility. The lower panels display the ratio of the data to the result of the signal plus background fit, with the hatched area showing the uncertainty in the combined fit

**Table 2:** Expected and observed ${\text {Z}{}{}} {\text {Z}{}{}}$ and ${\text {Z}{}{}} {\text {H}{}{}}$ signal strengths and their corresponding 95% CL upper limits. The expected signal strengths and the corresponding expected upper limits shown in parentheses include only the statistical uncertainties. The upper limits are obtained from a fit to the SvB signal probabilities under the hypothesis of no ${\text {Z}{}{}} {\text {Z}{}{}} \rightarrow 4{\text {b}{}{}}$ or ${\text {Z}{}{}} {\text {H}{}{}} \rightarrow 4{\text {b}{}{}}$ signal

	${\text {Z}{}{}} {\text {Z}{}{}}$	${\text {Z}{}{}} {\text {H}{}{}}$
Signal strength expected (stat. only)	$1.0 _{-1.7}^{+1.9}$ ($1.0 _{-1.3}^{+1.4}$)	$1.0 _{-1.4}^{+1.5}$ ($1.0 _{-1.1}^{+1.1}$)
Signal strength observed	$0.0 _{-1.7}^{+2.0}$	$2.2 _{-0.8}^{+0.9}$
Expected upper limit at $95\%$ CL (stat. only)	3.8 (2.8)	2.9 (2.3)
Observed upper limit at $95\%$ CL	3.8	5.0

The systematic uncertainty in the signal yields and distributions due to pileup is found to be negligibly small.

## Results

The measured and expected ${\text {Z}{}{}} {\text {Z}{}{}}$ and ${\text {Z}{}{}} {\text {H}{}{}}$ signal strengths are reported in terms of the signal strength modifier $\mu$, defined as the ratio of the value of the cross section to the expected SM theoretical cross section, $\sigma /\sigma _{\text {SM}}$. The $\text {CL}_\text {s}$ method [78, 79] is used to determine the upper limits on the signal strengths at 95% confidence level (CL).

The final fit procedure is validated using synthetic data samples without statistical fluctuations, and also by treating one of the mixed models as the observed four-tag data. In both tests, the behavior of the systematic uncertainties is as expected and the resulting best fit signal strengths were consistent with zero.

Figure 12 shows the results of the combined fit of the SvB signal probability distribution to the signal plus background model. The resulting signal strengths and corresponding 95% CL upper limits are shown in Table 2. The values if only the statistical uncertainties are included are shown in parentheses. The upper limits are determined under the assumption that no signal exists. Despite the significant difference in the ${\text {Z}{}{}} {\text {Z}{}{}}$ and ${\text {Z}{}{}} {\text {H}{}{}}$ cross sections, the upper limits of the signal strengths are similar. This is due to the increased signal selection efficiency and lower background levels in the SR for the ${\text {Z}{}{}} {\text {H}{}{}}$ channel in comparison to those for the ${\text {Z}{}{}} {\text {Z}{}{}}$ channel. The current results are limited by the size of the data set and the systematic uncertainties associated with the background model.

## Summary

A search for ${\text {Z}{}{}} {\text {Z}{}{}}$ and ${\text {Z}{}{}} {\text {H}{}{}}$ production in the 4b final state is presented. The search uses the full 2016–2018 data set of proton-proton collisions at a center-of-mass energy of 13$\,\text {Te}\hspace{-.08em}\text {V}$ recorded with the CMS detector at the LHC, corresponding to an integrated luminosity of 133$\,\text {fb}^{-1}$. The analysis benefits from a multiclass multivariate classifier, which uses convolutions to solve the combinatoric jet pairing problem, and has been designed with an architecture customized to the 4b final state. The classifier is used both for signal-versus-background discrimination and for the derivation and validation of the background model. A novel technique for assessing the background modeling uncertainties, using a synthetic data sample, produced using a hemisphere mixing procedure, allows both the uncertainty in the background model and its variance to be measured with a precision better than the statistical uncertainties in the selected signal-region events. While these techniques are developed and demonstrated in the ${\text {Z}{}{}} {\text {Z}{}{}}$ and ${\text {Z}{}{}} {\text {H}{}{}}$ $\rightarrow$ 4b searches, they are directly applicable to the ${\text {H}{}{}} {\text {H}{}{}} \rightarrow 4{\text {b}{}{}}$ analysis. The observed (expected) 95% CL upper limits on the ${\text {Z}{}{}} {\text {Z}{}{}} \rightarrow 4{\text {b}{}{}}$ and ${\text {Z}{}{}} {\text {H}{}{}} \rightarrow 4{\text {b}{}{}}$ production cross sections correspond to 3.8 (3.8) and 5.0 (2.9) times the standard model prediction, respectively.

## Data Availability

This manuscript has no associated data. [Author’s comment: Release and preservation of data used by the CMS Collaboration as the basis for publications is guided by the https://cms-docdb.cern.ch/cgi-bin/PublicDocDB/RetrieveFile?docid=6032 &filename=CMSDataPolicyV1.2.pdf &version=2  CMS data preservation, re-use and open access policy.]

## Code Availability Statement

This manuscript has no associated code/software. [Author’s comment: We do not supply a code availability statement.]

## References

[1] M. Cepeda et al., Report from working group 2: Higgs physics at the HL-LHC and HE-LHC. CERN Yellow Rep. Monogr. **7**, 221 (2019). 10.23731/CYRM-2019-007.221. arXiv:1902.00134 10.23731/CYRM-2019-007.221

[2] B.D. Micco, M. Gouzevitch, J. Mazzitelli, C. Vernieri, Higgs boson potential at colliders: status and perspectives. Rev. Phys. **5**, 100045 (2020). 10.1016/j.revip.2020.100045. arXiv:1910.00012 10.1016/j.revip.2020.100045

[3] CMS Collaboration, A portrait of the Higgs boson by the CMS experiment ten years after the discovery. Nature **607**, 60 (2022). 10.1038/s41586-022-04892-x. arXiv:2207.00043 10.1038/s41586-022-04892-x PMC9259501 35788190

[4] CMS Collaboration, Search for Higgs boson pair production in the four b quark final state in proton-proton collisions at . Phys. Rev. Lett. **129**, 081802 (2022). 10.1103/PhysRevLett.129.081802. arXiv:2202.09617 10.1103/PhysRevLett.129.081802 36053704

[5] ATLAS Collaboration, Search for Higgs boson pair production in the two bottom quarks plus two photons final state in pp collisions at TeV with the ATLAS detector. Phys. Rev. D ** 106**, 052001 (2022). 10.1103/PhysRevD.106.052001. arXiv:2112.11876

[6] CMS Collaboration, Search for nonresonant Higgs boson pair production in final state with two bottom quarks and two tau leptons in proton-proton collisions at . Phys. Lett. B **842**, 137531 (2023). 10.1016/j.physletb.2022.137531. arXiv:2206.09401

[7] ATLAS Collaboration, Search for resonant and non-resonant Higgs boson pair production in the decay channel using 13 TeV pp collision data from the ATLAS detector. JHEP ** 07**, 040 (2023). 10.1007/JHEP07(2023)040. arXiv:2209.10910

[8] CMS Collaboration, Search for nonresonant Higgs boson pair production in final states with two bottom quarks and two photons in proton-proton collisions at . JHEP **03**, 257 (2021). 10.1007/JHEP03(2021)257. arXiv:2011.12373

[9] ATLAS Collaboration, Search for nonresonant pair production of Higgs bosons in the final state in pp collisions at with the ATLAS detector. Phys. Rev. D **108**, 052003 (2023). 10.1103/PhysRevD.108.052003. arXiv:2301.03212

[10] CMS Collaboration, Search for nonresonant pair production of highly energetic Higgs bosons decaying to bottom quarks. Phys. Rev. Lett. **131**, 041803 (2023). 10.1103/PhysRevLett.131.041803. arXiv:2205.06667 10.1103/PhysRevLett.131.041803 37566864

[11] ATLAS Collaboration, Search for pair production of Higgs bosons in the final state using proton-proton collisions at TeV with the ATLAS detector. JHEP **01**, 030 (2019). 10.1007/JHEP01(2019)030. arXiv:1804.06174

[12] CMS Collaboration, Search for resonant pair production of Higgs bosons decaying to bottom quark-antiquark pairs in proton-proton collisions at 13 TeV. JHEP **08**, 152 (2018). 10.1007/JHEP08(2018)152. arXiv:1806.03548

[13] ATLAS Collaboration, Search for pair production of Higgs bosons in the final state using proton–proton collisions at TeV with the ATLAS detector. Phys. Rev. D ** 94**, 052002 (2016). 10.1103/PhysRevD.94.052002. arXiv:1606.04782

[14] ATLAS Collaboration, Search for Higgs boson pair production in the final state from pp collisions at TeV with the ATLAS detector. Eur. Phys. J. C **75**, 412 (2015). 10.1140/epjc/s10052-015-3628-x. arXiv:1506.00285 10.1140/epjc/s10052-015-3628-x PMC4564859 26380565

[15] CMS Collaboration, Search for resonant pair production of Higgs bosons decaying to two bottom quark-antiquark pairs in proton-proton collisions at 8 TeV. Phys. Lett. B **749**, 560 (2015). 10.1016/j.physletb.2015.08.047. arXiv:1503.04114

[16] E.D.L. Cren, A note on the history of mark-recapture population estimates. J. Anim. Ecol. **34**, 453 (1965). 10.2307/2661 10.2307/2661

[17] CDF Collaboration, Measurement of and in collisions at GeV. Phys. Rev. D **44**, 29 (1991). 10.1103/PhysRevD.44.29

[18] O. Behnke, K. Krőninger, G. Schott, T. Schőrner-Sadenius, Data Analysis in High Energy Physics: A Practical Guide to Statistical Methods. Wiley-VCH, Weinheim (2013). 10.1002/9783527653416

[19] P. De Castro Manzano et al., Hemisphere mixing: a fully data-driven model of QCD multijet backgrounds for LHC searches. PoS **EPS-HEP2017**, 370 (2017). 10.22323/1.314.0370. arXiv:1712.02538

[20] CMS Collaboration, Search for nonresonant Higgs boson pair production in the final state at 13 TeV. JHEP **04**, 112 (2019). 10.1007/JHEP04(2019)112. arXiv:1810.11854

[21] CMS Collaboration, Measurement of the ZZ production cross section and Z branching fraction in pp collisions at . Phys. Lett. B **763**, 280 (2016). 10.1016/j.physletb.2016.10.054. arXiv:1607.08834

[22] LHC Higgs Cross Section Working Group, Handbook of LHC Higgs cross sections: 4. Deciphering the nature of the Higgs sector, CERN Report CERN-2017-002-M (2016). 10.23731/CYRM-2017-002. arXiv:1610.07922

[23] CMS Collaboration, Measurements of production cross sections and constraints on anomalous triple gauge couplings at . Eur. Phys. J. C **81**, 200 (2021). 10.1140/epjc/s10052-020-08817-8. arXiv:2009.01186 10.1140/epjc/s10052-020-08817-8 PMC7921081 33750993

[24] ATLAS Collaboration, cross-section measurements and search for anomalous triple gauge couplings in 13 TeV pp collisions with the ATLAS detector. Phys. Rev. D ** 97**, 032005 (2018). 10.1103/PhysRevD.97.032005. arXiv:1709.07703

[25] CMS Collaboration, Observation of Higgs boson decay to bottom quarks. Phys. Rev. Lett. **121**, 121801 (2018). 10.1103/PhysRevLett.121.121801. arXiv:1808.08242 10.1103/PhysRevLett.121.121801 30296133

[26] ATLAS Collaboration, Observation of decays and VH production with the ATLAS detector. Phys. Lett. B **786**, 59 (2018). 10.1016/j.physletb.2018.09.013. arXiv:1808.08238

[27] C.M.S. Collaboration, Measurement of simplified template cross sections of the Higgs boson produced in association with W or Z bosons in the H decay channel in proton-proton collisions at = 13 TeV. Phys. Rev. D **109**, 092011 (2024). 10.1103/PhysRevD.109.092011 10.1103/PhysRevD.109.092011

[28] HEPData record for this analysis (2024). 10.17182/hepdata.146898

[29] CMS Collaboration, The CMS experiment at the CERN LHC. JINST **3**, S08004 (2008). 10.1088/1748-0221/3/08/S08004

[30] CMS Collaboration, Development of the CMS detector for the CERN LHC Run 3 (2023). arXiv:2309.05466

[31] CMS Collaboration, Performance of the CMS Level-1 trigger in proton-proton collisions at . JINST **15**, P10017 (2020). 10.1088/1748-0221/15/10/P10017. arXiv:2006.10165

[32] CMS Collaboration, The CMS trigger system. JINST **12**, P01020 (2017). 10.1088/1748-0221/12/01/P01020. arXiv:1609.02366

[33] CMS Collaboration, Electron and photon reconstruction and identification with the CMS experiment at the CERN LHC. JINST **16**, P05014 (2021). 10.1088/1748-0221/16/05/P05014. arXiv:2012.06888

[34] CMS Collaboration, Performance of the CMS muon detector and muon reconstruction with proton-proton collisions at 13 TeV. JINST **13**, P06015 (2018). 10.1088/1748-0221/13/06/P06015. arXiv:1804.04528

[35] CMS Collaboration, Description and performance of track and primary-vertex reconstruction with the CMS tracker. JINST **9**, P10009 (2014). 10.1088/1748-0221/9/10/P10009. arXiv:1405.6569

[36] CMS Collaboration, Particle-flow reconstruction and global event description with the CMS detector. JINST **12**, P10003 (2017). 10.1088/1748-0221/12/10/P10003. arXiv:1706.04965

[37] CMS Collaboration, Technical proposal for the Phase-II upgrade of the Compact Muon Solenoid, CMS Technical Proposal CERN-LHCC-2015-010, CMS-TDR-15-02, CERN (2015)

[38] M. Cacciari, G.P. Salam, G. Soyez, The anti- jet clustering algorithm. JHEP **04**, 063 (2008). 10.1088/1126-6708/2008/04/063. arXiv:0802.1189 10.1088/1126-6708/2008/04/063

[39] M. Cacciari, G.P. Salam, G. Soyez, FastJet user manual. Eur. Phys. J. C **72**, 1896 (2012). 10.1140/epjc/s10052-012-1896-2. arXiv:1111.6097 10.1140/epjc/s10052-012-1896-2

[40] C.M.S. Collaboration, Pileup mitigation at CMS in data. JINST **15**, P09018 (2020). 10.1088/1748-0221/15/09/p09018. arXiv:2003.00503 10.1088/1748-0221/15/09/p09018

[41] C.M.S. Collaboration, Jet energy scale and resolution in the CMS experiment in proton-proton collisions at . JINST **12**, P02014 (2017). 10.1088/1748-0221/12/02/P02014. arXiv:1607.03663 10.1088/1748-0221/12/02/P02014

[42] CMS Collaboration, Jet energy scale and resolution measurement with Run-2 legacy data collected by CMS at , CMS Detector Performance Summary CMS-DP-2021-033, CERN (2021)

[43] E. Bols et al., Jet flavour classification using DeepJet. JINST **15**, P12012 (2020). 10.1088/1748-0221/15/12/P12012. arXiv:2008.10519 10.1088/1748-0221/15/12/P12012

[44] C.M.S. Collaboration, Precision luminosity measurement in proton-proton collisions at 13 TeV in 2015 and 2016 at CMS. Eur. Phys. J. C **81**, 800 (2021). 10.1140/epjc/s10052-021-09538-2. arXiv:2104.01927 34781320 10.1140/epjc/s10052-021-09538-2 PMC8550658

[45] CMS Collaboration, CMS luminosity measurement for the 2017 data-taking period at , CMS Physics Analysis Summary CMS-PAS-LUM-17-004, CERN (2017)

[46] CMS Collaboration, CMS luminosity measurement for the 2018 data-taking period at , CMS Physics Analysis Summary CMS-PAS-LUM-18-002, CERN (2019)

[47] CMS Collaboration, Identification of heavy-flavour jets with the CMS detector in pp collisions at 13 TeV. JINST **13**, P05011 (2018). 10.1088/1748-0221/13/05/P05011. arXiv:1712.07158

[48] S. Frixione, P. Nason, G. Ridolfi, A positive-weight next-to-leading-order Monte Carlo for heavy flavour hadroproduction. JHEP **09**, 126 (2007). 10.1088/1126-6708/2007/09/126. arXiv:0707.3088 10.1088/1126-6708/2007/09/126

[49] S. Frixione, P. Nason, C. Oleari, Matching NLO QCD computations with parton shower simulations: the POWHEG method. JHEP **11**, 070 (2007). 10.1088/1126-6708/2007/11/070. arXiv:0709.2092 10.1088/1126-6708/2007/11/070

[50] J.M. Campbell, R.K. Ellis, P. Nason, E. Re, Top-pair production and decay at NLO matched with parton showers. JHEP **04**, 114 (2015). 10.1007/jhep04(2015)114. arXiv:1412.1828 10.1007/jhep04(2015)114

[51] J. Alwall et al., The automated computation of tree-level and next-to-leading order differential cross sections, and their matching to parton shower simulations. JHEP **07**, 79 (2014). 10.1007/jhep07(2014)079. arXiv:1405.0301 10.1007/jhep07(2014)079

[52] R. Frederix, S. Frixione, Merging meets matching in MC@NLO. JHEP **12**, 061 (2012). 10.1007/JHEP12(2012)061. arXiv:1209.6215 10.1007/JHEP12(2012)061

[53] K. Mimasu, V. Sanz, C. Williams, Higher order QCD predictions for associated Higgs production with anomalous couplings to gauge bosons. JHEP **08**, 039 (2016). 10.1007/JHEP08(2016)039. arXiv:1512.02572 10.1007/JHEP08(2016)039

[54] K. Hamilton, P. Nason, G. Zanderighi, MINLO: multi-scale improved NLO. JHEP **10**, 155 (2012). 10.1007/JHEP10(2012)155. arXiv:1206.3572 10.1007/JHEP10(2012)155

[55] G. Luisoni, P. Nason, C. Oleari, F. Tramontano, HW/HZ + 0 and 1 jet at NLO with the POWHEG BOX interfaced to GoSam and their merging within MiNLO. JHEP **10**, 083 (2013). 10.1007/JHEP10(2013)083. arXiv:1306.2542 10.1007/JHEP10(2013)083

[56] A. Djouadi, J. Kalinowski, M. Mühlleitner, M. Spira, Hdecay: Twenty++ years after. Comput. Phys. Commun. **238** (2019). 10.1016/j.cpc.2018.12.010

[57] Particle Data Group, R.L. Workman et al., Review of particle physics. Prog. Theor. Exp. Phys. **2022**, 083C01 (2022). 10.1093/ptep/ptac097

[58] G. Heinrich et al., NLO predictions for Higgs boson pair production with full top quark mass dependence matched to parton showers. JHEP **08**, 088 (2017). 10.1007/JHEP08(2017)088. arXiv:1703.09252 10.1007/JHEP08(2017)088

[59] S. Dawson, S. Dittmaier, M. Spira, Neutral Higgs boson pair production at hadron colliders: QCD corrections. Phys. Rev. D **58**, 115012 (1998). 10.1103/PhysRevD.58.115012. arXiv:hep-ph/9805244 10.1103/PhysRevD.58.115012

[60] S. Borowka et al., Higgs boson pair production in gluon fusion at next-to-leading order with full top-quark mass dependence. Phys. Rev. Lett. **117**, 012001 (2016). 10.1103/PhysRevLett.117.079901. arXiv:1604.06447. [Erratum: 10.1103/PhysRevLett.117.079901] 10.1103/PhysRevLett.117.012001 27419563

[61] J. Baglio et al., Gluon fusion into Higgs pairs at NLO QCD and the top mass scheme. Eur. Phys. J. C **79**, 459 (2019). 10.1140/epjc/s10052-019-6973-3. arXiv:1811.05692 31258411 10.1140/epjc/s10052-019-6973-3 PMC6560654

[62] D. de Florian, J. Mazzitelli, Higgs boson pair production at next-to-next-to-leading order in QCD. Phys. Rev. Lett. **111**, 201801 (2013). 10.1103/PhysRevLett.111.201801. arXiv:1309.6594 24289675 10.1103/PhysRevLett.111.201801

[63] D.Y. Shao, C.S. Li, H.T. Li, J. Wang, Threshold resummation effects in Higgs boson pair production at the LHC. JHEP **07**, 169 (2013). 10.1007/JHEP07(2013)169. arXiv:1301.1245 10.1007/JHEP07(2013)169

[64] D. de Florian, J. Mazzitelli, Higgs pair production at next-to-next-to-leading logarithmic accuracy at the LHC. JHEP **09**, 053 (2015). 10.1007/JHEP09(2015)053. arXiv:1505.07122 10.1007/JHEP09(2015)053

[65] M. Grazzini et al., Higgs boson pair production at NNLO with top quark mass effects. JHEP **05**, 059 (2018). 10.1007/JHEP05(2018)059. arXiv:1803.02463 10.1007/JHEP05(2018)059

[66] J. Baglio et al., : combined uncertainties. Phys. Rev. D **103**, 056002 (2021). 10.1103/PhysRevD.103.056002. arXiv:2008.11626 10.1103/PhysRevD.103.056002

[67] T. Sjöstrand et al., An introduction to pythia 8.2. Comput. Phys. Commun. **191**, 159 (2015), 10.1016/j.cpc.2015.01.024. arXiv:1410.3012

[68] CMS Collaboration, Extraction and validation of a new set of CMS pythia8 tunes from underlying-event measurements. Eur. Phys. J. C **80**, 4 (2020). 10.1140/epjc/s10052-019-7499-4. arXiv:1903.12179 10.1140/epjc/s10052-019-7499-4 PMC6944267 31976986

[69] CMS Collaboration, Event generator tunes obtained from underlying event and multiparton scattering measurements. Eur. Phys. J. C **76**, 155 (2016). 10.1140/epjc/s10052-016-3988-x. arXiv:1512.00815 10.1140/epjc/s10052-016-3988-x PMC4946872 27471433

[70] NNPDF Collaboration, Parton distributions for the LHC Run II. JHEP **04**, 040 (2015). 10.1007/JHEP04(2015)040. arXiv:1410.8849

[71] R.D. Ball et al., Parton distributions from high-precision collider data. Eur. Phys. J. C **77**, 663 (2017). 10.1140/epjc/s10052-017-5199-5. arXiv:1706.00428 31997920 10.1140/epjc/s10052-017-5199-5 PMC6956957

[72] GEANT4 Collaboration, GEANT4—a simulation toolkit. Nucl. Instrum. Meth. A **506**, 250 (2003). 10.1016/S0168-9002(03)01368-8

[73] CMS Collaboration, Evidence for the Higgs boson decay to a bottom quark–antiquark pair. Phys. Lett. B **780**, 501 (2018). 10.1016/j.physletb.2018.02.050. arXiv:1709.07497

[74] W. Shang, K. Sohn, D. Almeida, H. Lee, Understanding and improving convolutional neural networks via concatenated rectified linear units, in *Proc. 33rd Int. Conf. on Machine Learning*, vol. 48 (PMLR, 2016). arXiv:1603.05201

[75] K. He, X. Zhang, S. Ren, J. Sun, Deep residual learning for image recognition, in * 2016 IEEE Conf. in Computer Vision and Pattern Recognition (CVPR)* (2016). arXiv:1512.03385. 10.1109/CVPR.2016.90

[76] A. Vaswani et al., Attention is all you need, in *Advances in Neural Information Processing Systems*, vol. 30 (Curran Associates, Inc., 2017). arXiv:1706.03762

[77] R.A. Fisher, On the interpretation of from contingency tables, and the calculation of . J. R. Stat. Soc. (1922). 10.2307/2340521

[78] T. Junk, Confidence level computation for combining searches with small statistics. Nucl. Instrum. Meth. A **434**, 435 (1999). 10.1016/S0168-9002(99)00498-2. arXiv:hep-ex/9902006 10.1016/S0168-9002(99)00498-2

[79] A.L. Read, Presentation of search results: the CL technique. J. Phys. G **28**, 2693 (2002). 10.1088/0954-3899/28/10/313 10.1088/0954-3899/28/10/313

